# Paracrine Factors of Stressed Peripheral Blood Mononuclear Cells Activate Proangiogenic and Anti-Proteolytic Processes in Whole Blood Cells and Protect the Endothelial Barrier

**DOI:** 10.3390/pharmaceutics14081600

**Published:** 2022-07-30

**Authors:** Dragan Copic, Martin Direder, Klaudia Schossleitner, Maria Laggner, Katharina Klas, Daniel Bormann, Hendrik Jan Ankersmit, Michael Mildner

**Affiliations:** 1 Department of Thoracic Surgery, Medical University of Vienna, 1090 Vienna, Austria; dragan.copic@meduniwien.ac.at (D.C.); martin.direder@meduniwien.ac.at (M.D.); laggner.maria@gmail.com (M.L.); katharina.klas@meduniwien.ac.at (K.K.); daniel.bormann@meduniwien.ac.at (D.B.); 2 Laboratory for Cardiac and Thoracic Diagnosis and Regeneration, Department of Thoracic Surgery, Medical University of Vienna, 1090 Vienna, Austria; 3 Skin and Endothelium Research Division, Department of Dermatology, Medical University of Vienna, 1090 Vienna, Austria; klaudia.schossleitner@meduniwien.ac.at; 4 Department of Dermatology, Medical University of Vienna, 1090 Vienna, Austria

**Keywords:** regenerative medicine, cell-free secretomes, paracrine factors, single-cell RNA sequencing, serine protease inhibitor, endothelial barrier

## Abstract

Tissue-regenerative properties have been attributed to secreted paracrine factors derived from stem cells and other cell types. In particular, the secretome of γ-irradiated peripheral blood mononuclear cells (PBMCsec) has been shown to possess high tissue-regenerative and proangiogenic capacities in a variety of preclinical studies. In light of future therapeutic intravenous applications of PBMCsec, we investigated the possible effects of PBMCsec on white blood cells and endothelial cells lining the vasculature. To identify changes in the transcriptional profile, whole blood was drawn from healthy individuals and stimulated with PBMCsec for 8 h ex vivo before further processing for single-cell RNA sequencing. PBMCsec significantly altered the gene signature of granulocytes (17 genes), T-cells (45 genes), B-cells (72 genes), and, most prominently, monocytes (322 genes). We detected a strong upregulation of several tissue-regenerative and proangiogenic cyto- and chemokines in monocytes, including *VEGFA*, *CXCL1*, and *CXCL5*. Intriguingly, inhibitors of endopeptidase activity, such as *SERPINB2*, were also strongly induced. Measurement of the trans-endothelial electrical resistance of primary human microvascular endothelial cells revealed a strong barrier-protective effect of PBMCsec after barrier disruption. Together, we show that PBMCsec induces angiogenic and proteolytic processes in the blood and is able to attenuate endothelial barrier damage. These regenerative properties suggest that systemic application of PBMCsec might be a promising novel strategy to restore damaged organs.

## 1. Introduction

Central goals in modern regenerative medicine are the repair of injured tissues and organs along with restoration of their innate functions [1]. Cell-based therapies have made tangible progress in this field over the past decades [2]. Mesenchymal stem cells (MSC) possess high capacities for self-renewal and differentiation, which makes them a highly attractive option to promote tissue regeneration. However, in spite of encouraging pre-clinical data, most of the first-in-men clinical trials failed to show effectivity in patients [3,4]. Beyond that, it became increasingly apparent that released paracrine factors, rather than engraftment and differentiation of applied stem cells, are crucial for most of the beneficial biological effects and, thus, predominantly contribute to the observed tissue-regenerative effects [5,6,7,8]. In addition, stem-cell secretome-based therapies bear considerable limitations, including the need for invasive procedures for isolation, low abundance, and high costs for expansion and preservation. This highlights the need for alternative sources [9].

Recently, several studies suggested peripheral blood mononuclear cells (PBMCs) as a valuable alternative source to MSCs [10,11]. In addition, γ-irradiation of PBMCs has been shown to promote the production and release of soluble factors and to exert tissue protection [10]. In-depth functional analyses of the secretome of γ-irradiated PBMCs (PBMCsec) revealed a vast array of proteins, lipids, and extracellular vesicles as the main biological constituents and confirmed that the presence of all fractions is required in order for PBMCsec to exert its biological effects to full capacity [12,13]. The modes of action via which PBMCsec exerts its beneficial effects range from promotion of angiogenesis [12], antimicrobial activity [14], cytoprotection [15], immunomodulation [16], improvement of re-epithelization [17] to vasodilation and the inhibition of platelet aggregation [18]. More recently, Laggner and colleagues were able to demonstrate a decrease of dendritic cell-mediated skin inflammation in a murine model of contact hypersensitivity, as well as inhibition of basophil and mast cell degranulation after treatment with PBMCsec, further expanding the broad spectrum of possible clinical implications of this investigational medicinal product [19,20]. Beneficial effects of PBMCsec have thus far been examined in numerous preclinical setups of experimental tissue damage, including acute myocardial infarction, autoimmune myocarditis, stroke, and spinal cord injury (for a review, see [21]). Topical administration of PBMCsec enhanced wound healing in a murine full-thickness skin model [17] and improved tissue survival in a rodent epigastric flap model [22]. Similar results were observed in a porcine model of burn injury, where the application of PBMCsec markedly improved epidermal thickness after injury [23]. In addition, beneficial effects of PBMCsec were also observed after intraperitoneal or intravenous administration. PBMCsec decreased the affected area and improved neurological outcome in rodent models of cerebral ischemia and acute spinal cord injury after systemic application [24,25]. Furthermore, PBMCsec ameliorated myocardial damage, improved overall cardiac performance, and promoted cytoprotection of cardiomyocytes in rodent and porcine models of acute myocardial infarction (AMI) [10,15]. Interestingly, transcriptional changes were not restricted to the infarcted myocardium and the circumjacent heart tissue but also detected in the liver and the spleen, suggesting a systemic effect of PBMCsec beyond the site of injury [26].

For the treatment of cardiovascular pathologies caused by thromboembolic occlusion of arterial blood flow such as acute myocardial infarction and ischemic stroke, the rapid interventional re-establishment of vessel perfusion still remains the first-line therapy as it maximizes the rescue of ischemic tissue and, thus, decisively determines the primary outcome in affected patients [27]. However, increasing emphasis is attributed to the attenuation of damages secondary to reperfusion injury, as it represents a considerable risk factor for long-term tissue functions [28]. During extended periods of oxygen deprivation, cells suffer from intracellular calcium overload [29] and disturbed mitochondrial energy production [30], which ultimately lead to cell death and the release of inflammatory mediators and reactive oxygen species [31]. In turn, inflammatory cells infiltrate the damaged area and release proinflammatory cyto- and chemokines. In addition, neutrophils release serine proteases [32,33], which contribute to endothelial barrier dysfunction, further enhancing vascular injury in small arterial blood vessels and downstream capillaries [34]. As a result, large amounts of intravascular fluids along with the damaging mediators diffuse into the interstitial space to cause further damage [34].

Since PBMCsec has shown tissue-regenerative properties in several animal models and there is an urgent need for novel systemic tissue-regenerative therapeutic interventions, we investigated the effects of PBMCsec on white blood cells and on the endothelial barrier function.

## 2. Materials and Methods

### 2.1. Ethics Statement

This study was conducted in accordance with the Declaration of Helsinki and local regulations. Blood samples were obtained from healthy volunteers who had given their consent to donate. Use of primary HUVECs, primary DMECs, and blood samples was approved by the Institutional Review Board of the Medical University of Vienna (Ethics committee votes: 1280/2015, 1539/2017, and 1621/2020). All donors provided written informed consent.

### 2.2. Generation of PBMCsec

**Figure 1:**
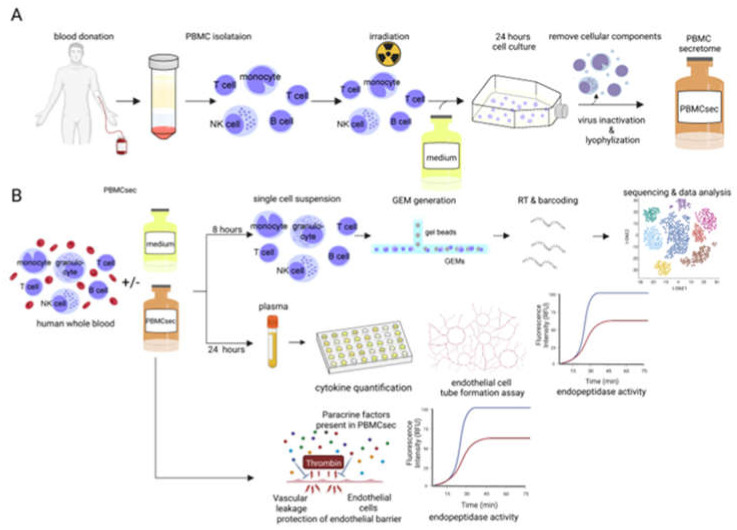
Experimental overview (**A**) Isolation of PBMCs from healthy donors and subsequent procedures necessary for the generation of PBMCsec. (**B**) The experimental setup involved two branches. In order to investigate pharmacodynamics effects of PBMCsec, human whole blood cells were treated with PBMCsec or left untreated for 8 h prior to further processing them for single-cell RNA sequencing. Plasma was collected from PBMCsec-treated whole blood and controls after 24 h of incubation and analyzed in a series of functional assays. In addition, we evaluated effects mediated directly by PBMCsec on endopeptidase activity and endothelial barrier protection. This figure was generated using Biorender.com (accessed on 6 June 2022).

Isolation of PBMCs and generation of PBMCsec have previously been described in detail [35], and a graphical overview is given in Figure 1A. In brief, PBMCs of volunteer blood donors aged 18–45 years were enriched by Ficoll-Paque PLUS (GE Healthcare, Chicago, IL, USA) density centrifugation, diluted to a concentration of 2.5 × 10^7^ cells/mL in CellGenix granulocyte–monocyte progenitor dendritic cell medium (CellGenix, Freiburg, Germany) and exposed to 60 Gy cesium-137 γ-irradiation (IBL 437C, Isotopen Diagnostik CIS GmbH, Dreieich, Germany). Following 24 h of incubation, cells and cellular debris were removed by centrifugation at 800× *g* for 15 min, and supernatants were passed through a 0.2 mm filter. The cell-free secretome generated by 2.5 × 10^7^ cells/mL corresponds to 25 units/mL PBMCsec. Next, methylene blue treatment was performed for viral clearance [35]. Secretomes were lyophilized, terminally sterilized by high-dosage-irradiation (Gammatron 1500, UKEM60Co irradiator with a maximum capacity of 1.5 MCi), and cryopreserved. Lyophilized compounds were reconstituted in 0.9% NaCl (B. Braun Melsungen AG, Melsungen, Germany) to the desired concentrations.

### 2.3. Preparation of Single-Cell Suspension of Human Whole Blood

For scRNA-seq, heparinized human whole blood was drawn from two age-matched male donors (age donor 1: 29 years; age donor 2: 29 years). A total of 3 mL of whole blood was either treated with PBMCsec (GMP APOSEC lot number: A00918399135; diluted in 0.9% NaCl; final concentration: 12.5 units/mL) or left untreated. Samples were incubated at 37 °C for 8 h. Red blood cells were removed by Red Blood Cell Lysis Buffer (Abcam, Cambridge, MA, USA). Cells were then washed twice with PBS containing 0.04% bovine serum albumin (BSA) and sequentially passed through 100 and 40 µm cell strainers. Using the LUNA-FL Dual Fluorescence Cell Counter (BioCat, Heidelberg, Germany) and the Acridine Orange/Propidium Iodide Cell Viability Kit (Logos Biosystems, Gyeonggi-do, South Korea), samples were set at a concentration of 1 × 10^6^ cells/mL and displayed a viability above 90%.

### 2.4. Dermal Microvascular Endothelial Cell Culture

Dermal microvascular endothelial cells (DMECs) were isolated from human foreskin. Foreskin was digested with dispase (Corning). The epidermis was removed, and the foreskin was scraped to dislodge endothelial cells. Cells were sorted for CD31 with magnetic beads (Thermo Fisher Scientific). Endothelial cells were cultured in endothelial growth medium (EGM-2; Lonza) containing 15% fetal bovine serum (FCS; Thermo Fisher Scientific, Waltham, MA, USA) and supplements for microvascular cells (Lonza). Cells were maintained in a humidified atmosphere containing 5% CO_2_ at 37 °C and passaged at 90% confluence. Prior to experiments, cells were authenticated and confirmed to be free of contamination by mycoplasma. Endothelial cells were used at passages 2–8.

### 2.5. Tube Formation Assay

Proangiogenic properties of PBMCsec and plasma of PBMCsec-treated whole blood were compared in a tube formation assay with human umbilical vein endothelial cells (HUVECs, passage 8) as described previously [36]. HUVECs (Lonza, Basel, Switzerland) were thawed and routinely cultured in polystyrene culture flasks (Merck Millipore, Burlington, MA, USA) containing endothelial cell basal medium-2 (EBM-2; Lonza) supplemented with endothelial cell growth medium-2 (EGM-2, Lonza) until fully confluent. The medium was changed every other day for a total of two passages. Prior to the tube formation assay, cells were maintained in EBM-2 containing 3% (*v*/*v*) heat-inactivated fetal bovine serum (Lonza) overnight and starved in basal EBM-2 without supplements for 3 h. Cells were seeded on growth factor-reduced Matrigel Matrix (Corning Inc. Life Sciences, Tewksbury, MA, USA) in µ-slides Angiogenesis (ibidi GmbH, Graefelfing, Germany) at a density of 10 × 10^4^ cells per well and stimulated with the supernatant obtained from 3 mL of whole blood cells for 4 h. Micrographs were acquired by an inverted phase contrast microscope (CKX41 Olympus Corporation; Tokyo, Japan) equipped with a 10× objective (CAch N, 10×/0.25 PhP; Olympus) using a SC30 camera (Olympus) and cellSens Entry software (version 1.8; Olympus). Tubule formation was quantified by the Angiogenesis Analyzer plugin [37] of ImageJ (version 1.53, java 1.8.0_172) using default settings.

### 2.6. Protein Quantification by Enzyme-Linked Immunosorbent Assay (ELISA)

For in vitro experiments, heparinized human whole blood was drawn from male donors (age donor 1:29 years; age donor 2:29 years; age donor 3:30 years). A total of 3 mL of whole blood was centrifuged (1000× *g* for 10 min at room temperature) freshly after venipuncture or after 24 h long cultivation of whole blood in absence or presence of 12.5 units/mL PBMCsec. Plasma samples were then stored at −20 °C until further use. Protein levels of Human CXCL1, human CXCL5, human SERPINB2, human VEGF-A, and human urokinase (R&D Systems, Biotechne, Minneapolis, MN, USA) were quantified by ELISA as recommended by the manufacturer. Absorbance was measured at 450 nm by a Spark multimode microplate reader (Tecan, Männedorf, Switzerland), and analyte quantifications were determined using external standard curves.

### 2.7. Protease Activity Assays

To test the inhibitory effects of PBMCsec on protease activity, we performed a fluorometric enzyme activity assay (Enzcheck) using the unselective serine protease trypsin (ThermoFisher Scientific, Waltham, MA, USA) at a concentration of 0.05%. Enzyme substrate was diluted in provided assay buffer according to manufacturer’s instruction. Equal amounts of trypsin were 1:2 diluted in assay buffer, control medium, or PBMCsec concentrated at 12.5 units/mL for 5 min before adding 10 µL to the prepared substrate mixture adding up to a total volume of 100 µL per well. The Urokinase Inhibitor Screening Kit (Sigma-Aldrich, St. Louis, MO, USA) was used to test the effect of the investigated plasma samples and PBMCsec on urokinase activity. In brief, 45 µL plasma sample were diluted in an equal volume of provided assay buffer. Human urokinase and substrate were added as suggested by the protocol, adding up to a total reaction volume of 100 µL per well. Samples from three donors were analyzed. For both tests, samples were then incubated at room temperature for a total of 60 min. Absorbance at 450 nm was measured by FluoStar Optima microplate reader (BMG Labtech, Ortenberg, Germany) in 15 min intervals.

### 2.8. Electrical Cell-Substrate Impedance Sensing (ECIS)

Electrical cell-substrate impedance sensing (ECIS, Applied Biophysics, Troy, NY, USA) was used to measure the electrical resistance of endothelial monolayers. A total of 12,000 endothelial cells were seeded on array plates (Ibidi) coated with gelatin (Sigma). After the resistance at 4000 Hz reached a stable plateau of >1000 Ω, endothelial cells were treated with indicated substances. Electrical resistance of cell monolayers was continuously monitored at 250 Hz [38].

### 2.9. Gel Bead-In Emulsion (GEMs) and Library Preparation

Single-cell RNA-seq was performed using the 10X Genomics Chromium Single-Cell Controller (10X Genomics, Pleasanton, CA, USA) with the Chromium Single-Cell 3′ V3 Kit following the manufacturer’s instructions. After quality control, RNA sequencing was performed by the Biomedical Sequencing Core Facility of the Center for Molecular Medicine (Center for Molecular Medicine, Vienna, Austria) on an Illumina HiSeq 3000/4000 (Illumina, San Diego, CA, USA). For donor 1, we detected 2003 cells in the untreated sample and 1281 cells in the PBMCsec-treated sample, while donor 2 had 12,356 cells in the untreated sample and 10,865 cells in the PBMCsec-treated sample. Raw sequencing data were then processed with the Cell Ranger v3.0.2 software (10X Genomics, Pleasanton, CA, USA) for demultiplexing and alignment to a reference genome (GRCh38).

### 2.10. Data Analysis

Secondary data analysis was performed using R Studio in R (Version 4.0.4; The R Foundation, Vienna, Austria) using the R software package “Seurat” (Seurat v.4.0.0, Satija Lab, New York, NY, USA). Cells were first analyzed for their unique molecular identifiers (UMI) and mitochondrial gene counts to remove unwanted variations in the scRNA-seq data. Cells with UMI counts below 200 or above 2500 and more than 10% of mitochondrial genes were excluded from the dataset. Next, we followed the recommended standard workflow for integration of scRNA-seq datasets [39]. Data were scaled, and principal component analysis (PCA) was performed. Statistically significant principal components (PCs) were identified by visual inspection. Using the Louvain algorithm at a resolution of 0.025, we identified a total of four communities. The preselected PCs and identified clusters served for Uniform Manifold Approximation and Projection for Dimension Reduction (UMAP). After bioinformatics integration of datasets of untreated and PBMCsec-treated samples, erythrocytes were removed by excluding all cells with expression of hemoglobin subunit beta (HBB) >0.5. Clusters were then annotated on the basis of the expression of well-established cell-type-defining marker genes. We used UMAP-plots, feature plots, heat maps, volcano plots, and violin plots to visualize differences between the investigated conditions. To determine DEGs, normalized count numbers were used. We applied the FindMarkers argument using default settings to calculate DEGs for clusters of interest between conditions with a log-foldchange threshold of 0.25 and an adjusted *p*-value <0.05. A log_2_ fold-change increase in gene expression above 1 was considered as upregulation, while a decrease below −1 was considered as downregulation. Only genes with an avgLog_2_FC above 1 and below −1 were forwarded to the Metascape [40] online software package to identify significantly enriched pathways (−log_10_(*p*-value) >2). Additionally, the same gene sets were processed by Cytoscape plug-in ClueGO [41] to visualize significantly (*p*-value <0.05, kappa score: 0.4) enriched molecular functions for the investigated conditions.

### 2.11. Statistical Analysis

For single-cell RNA-seq, two donors were analyzed. Negative binomial regression was performed to normalize data and achieve variance stabilization. The Wilcoxon rank sum test was applied, followed by the Bonferroni post hoc test, to calculate differentially expressed genes. For in vitro experiments, at least three different donors were used. For data analysis of the tube formation assay, investigators were blinded to treatments. Data were statistically evaluated using GraphPad Prism v8.0.1 software (GraphPad Software, San Diego, CA, USA). When analyzing three or more groups, ordinary one-way ANOVA and multiple comparison post hoc tests with Dunnett’s correction were calculated, and *p*-values <0.05 were considered statistically significant. Data are presented as the mean ± standard error of the mean (SEM).

## 3. Results

### 3.1. PBMCsec Modulates the Gene Signature of T Cells, B Cells, Granulocytes, and Monocytes

**Figure 2:**
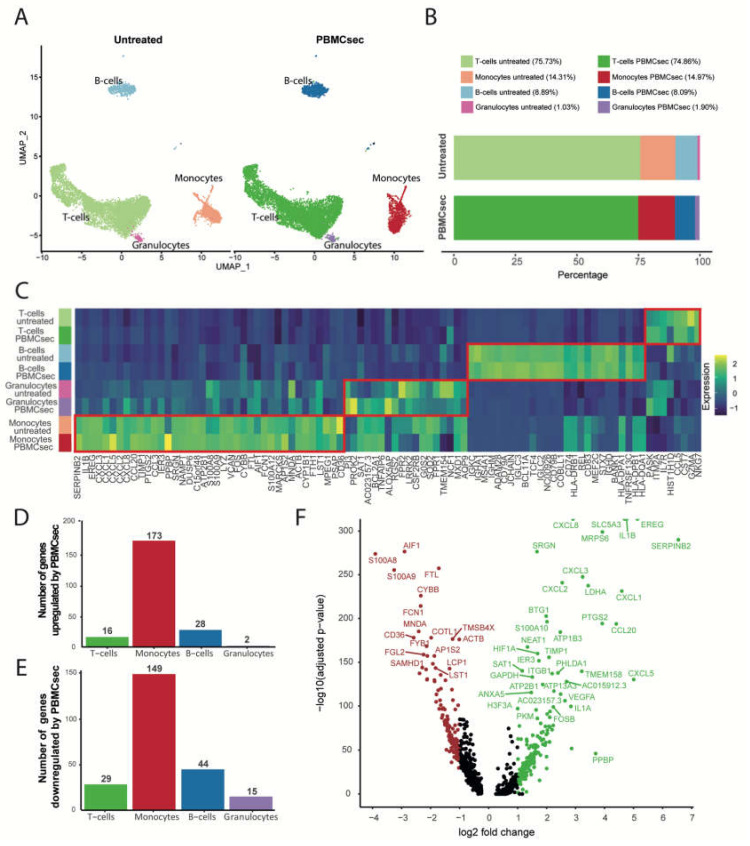
Ex vivo treatment of human whole blood cells alters the transcriptional profile in the monocyte subset and upregulates pathways associated with tissue regeneration. (**A**) UMAP clustering identified T-cells, monocytes, B-cells, and granulocytes in untreated and PBMCsec-treated samples with (**B**) similar cell frequency for each cluster between the investigated conditions. (**C**) Heatmap of cluster-defining marker genes of normalized gene expression showing distinct gene patterns between T-cells, monocytes, B-cells, and granulocytes. Bar plots represent the number of (**D**) up- and (**E**) downregulated genes in PBMCsec-treated samples compared to untreated control. Genes with an adjusted *p*-value < 0.05 and an average log_2_ fold change ≥1 or ≤−1 were considered as DEGs. (**F**) Volcano plot showing the regulated genes in monocytes treated with PBMCsec when compared to untreated monocytes. Upregulated genes are marked in green, while downregulated genes are shown in red.

To investigate the degree to which PBMCsec alters the transcriptional landscape of immune cells in human whole blood, we conducted single-cell RNA sequencing (scRNA-seq) of whole-blood samples treated ex vivo with PBMCsec for 8 h. A methodological overview of the experimental approach employed in this study is provided in Figure 1. Bioinformatics analysis and UMAP clustering revealed four main cell populations consisting of monocytes, T-cells, B-cells, and granulocytes in all investigated conditions (Figure 2A). Identification was based on the expression of cluster marker genes including the CD14 molecule (*CD14*), CD3D molecule (CD3D), membrane spanning four domains A1 (*MS4A1*), and peptidase inhibitor 3 (*PI3*) for the respective cell types (Figure S1). Although significantly fewer cells were analyzed from donor 1, all cell types were found in both donors (Figure S2A). Treatment with PBMCsec did not result in a significant change in relative cell numbers (percentage of cells in untreated vs PBMCsec for T-cells: 75.73% vs. 74.86%; monocytes: 14.31% vs. 14.97%; B-cells: 8.89% vs. 8.09%; granulocytes 1.03% vs. 1.90%) (Figure 2B). The transcriptional heterogeneity between cell types was confirmed in a heatmap showing the average expression of cluster-defining genes of each cell type (Figure 2C). Next, we assessed changes in gene expression for all cell populations after treatment with PBMCsec. We calculated the number and distribution of significantly up- (Figure 2D) and downregulated genes (Figure 2E) for each cell type compared to the untreated control. A total number of 45 differentially expressed genes (DEG) (16 upregulated; 29 downregulated) were detected in T-cells, along with 72 in B-cells (28 upregulated, 44 downregulated) and 17 in granulocytes (two upregulated; 15 downregulated) (Figure S3A–C). Monocytes displayed the highest number of differentially expresses genes (173 upregulated; 148 downregulated), including upregulation of serpin family B member 2 (SERPINB2), epiregulin (EREG), interleukin 1 beta (IL1B), C–X–C motif chemokine ligand 1 (CXCL1), C–X–C motif chemokine ligand 3 (CXCL3), (CXCL5), and vascular endothelial growth factor A (VEGFA), and downregulation of S100 calcium-binding protein A8 (S100A8), S100 calcium-binding protein A9 (S100A9), allograft inflammatory factor 1 (AIF1), CD36 molecule (CD36), and CD163 molecule (CD163), amongst others (Figure 2F). From this, we can conclude that ex vivo stimulation of human whole blood with PBMCsec significantly changed gene expression in blood immune cells, most prominently in monocytes.

### 3.2. PBMCsec Induces Tissue-Regenerative Pathways in Monocytes from Human Whole Blood

**Figure 3:**
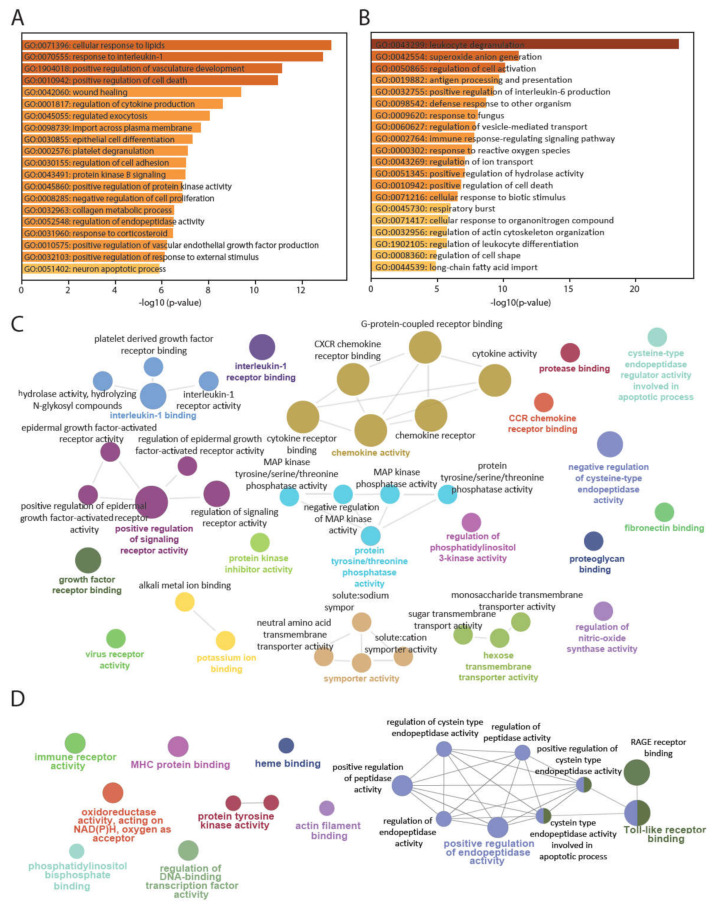
Tissue-regenerative pathways are upregulated in monocytes treated with PBMCsec Gene ontology pathway analysis was performed in Metascape. The top 20 biological processes affected by the upregulated (**A**) and downregulated (**B**) DEG in monocytes treated with PBMCsec are listed and hierarchically sorted by −log_10_(*p*-value) and enrichment factor (>2). (**C**) ClueGO visualization of molecular functions associated with upregulated and (**D**) downregulated genes in monocytes treated with PBMCsec.

Next, we identified biological pathways associated with the differentially regulated genes across the identified cell types. Biological functions such as angiogenesis and cytokine production were enriched in T- and B-cells treated with PBMCsec (Figure S3D,F), while activation of myeloid cells and responses to oxidative stress were associated with the downregulated gene sets (Figure S3E,G). No significantly regulated pathways were identified in granulocytes. The top Gene Ontology pathways associated with upregulated genes in monocytes after treatment with PBMCsec included response to lipid and to interleukin 1, along with terms strongly associated with wound healing, angiogenesis, regulation and production of cytokines, and regulation of endopeptidase activity (Figure 3A). Upregulated gene sets in monocytes treated with PBMCsec were highly comparable between the two donors (Figure S2B,C), and expression of *SERPINB2*, *VEGFA*, *CXCL1*, and *CXCL5* was significantly upregulated by PBMCsec in monocytes from both donors (Figure S2D,E), indicating a low donor variability. Major pathways associated with biological processes resulting from the downregulated gene set in monocytes treated with PBMCsec involved activation and differentiation of leukocytes, generation of reactive oxide species, and processing and presentation of antigens (Figure 3B). The complete lists of all up- and downregulated pathways (Figure S4A,B) and genes (Supplementary Files S1 and S2, respectively) are provided as Supplementary Materials. A closer look at the genes involved in the degranulation and cell activation of leukocytes revealed different members of the S100 and leukocyte immunoglobulin-like receptor (LILR) families to be downregulated in monocytes treated with PBMCsec (Figure S5A). Furthermore, we observed a downregulation of genes associated with the generation of reactive oxygen species, including the scavenger receptor CD36, the pattern recognition receptor CLEC7A, and a subunit of the NADPH oxidase complex in NCF1 (Figure S5B). We further sought to confirm our findings using ClueGO to investigate molecular functions related to these gene sets. In line with the initial analysis, we again identified strong associations with molecular functions related to cyto- and chemokine activity, signaling, and negative regulation of cysteine-type endopeptidase activity for the upregulated genes (Figure 3C), while functions related to immune receptor signaling, oxidoreductase activity, and antigen presentation were significantly associated with the set of downregulated genes (Figure 3D). From this analysis, we can conclude that monocytes were mostly affected by PBMCsec, resulting in the induction of tissue-regenerative processes, while processes associated with leukocyte activation and reactive oxygen species generation were downregulated.

### 3.3. Paracrine Factors in the Plasma of PBMCsec-Treated Whole Blood Exert Proangiogenetic Effects In Vitro

**Figure 4:**
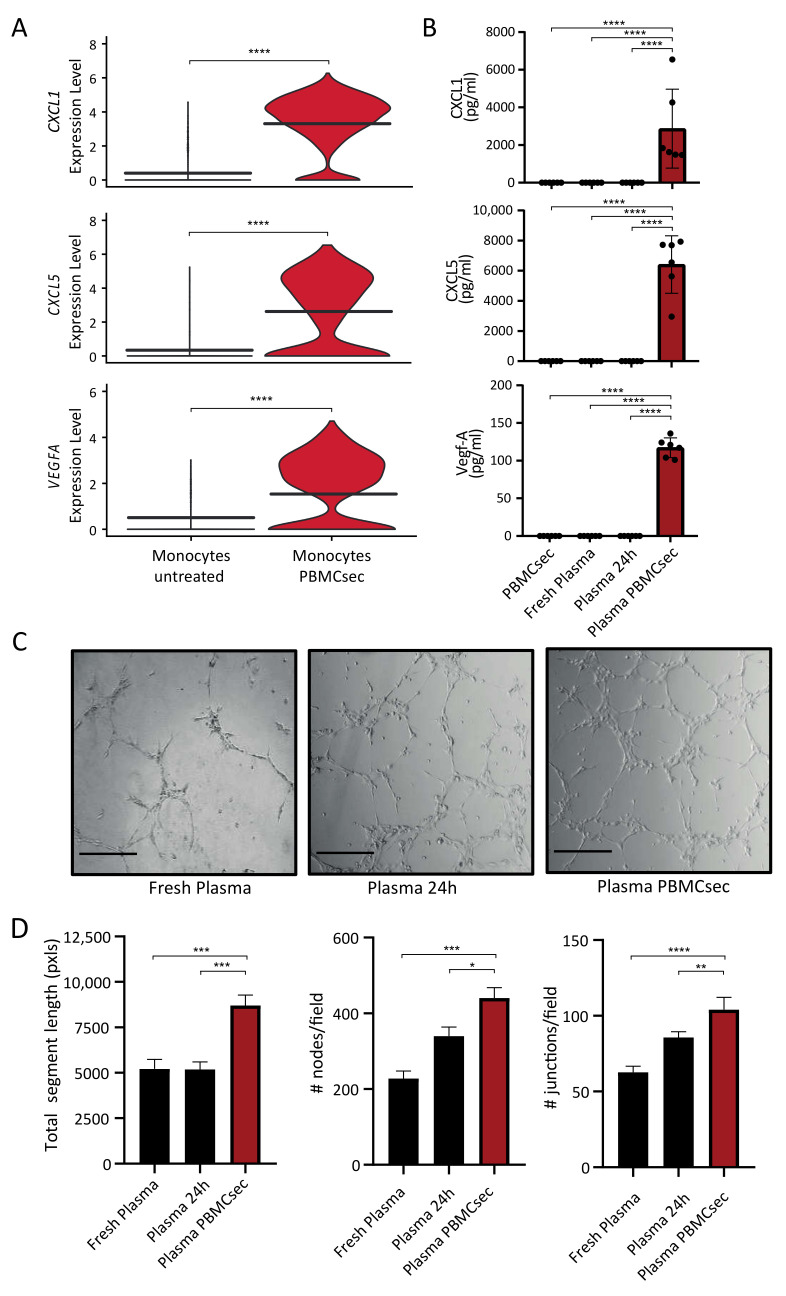
Paracrine factors are induced by PBMCsec in human plasma and enhance endothelial cell tube formation in vitro. (**A**) Gene expression of the proangiogenic chemokines *CXCL1* and *CXCL5*, as well as growth factor *VEGFA*, is strongly induced in monocytes treated with PBMCsec when compared to untreated monocytes (**B**) Assessment of protein levels for CXCL1, CXCL5, and VEGF-A by ELISA in plasma from PBMCsec-treated whole blood and controls (*n* = 3 biological replicates measured in duplicates). (**C**) Representative images of HUVEC tube formation assay in presence of fresh plasma, plasma from untreated whole blood, and plasma obtained from PBMCsec-treated whole blood after 24 h of ex vivo cultivation (scale bar 250 µm) with (**D**) analysis of number of nodes and junctions per field and total segment length (*n* = 3). Ordinary one-way ANOVA was performed. Dunnett’s multiple comparison test was carried out to compare groups; * *p* < 0.05, ** *p* < 0.01, *** *p* < 0.001, **** *p* < 0.0001.

Since several cytokines and growth factors were upregulated in monocytes treated with PBMCsec (Figure 2F), we next assessed the protein levels of selected factors in plasma derived from PBMCsec-treated whole blood. PBMCsec, fresh plasma collected immediately after venipuncture, and untreated plasma after ex vivo incubation for 24 h served as controls. Gene expression of CXCL1, CXCL5, and VEGFA showed strong upregulation in monocytes after stimulation with PBMCsec (Figure 4A). This was confirmed on a protein level, when CXCL1 (2873 ± 2093 pg/mL), CXCL5 (6413 ± 1911 pg/mL), and VEGF-A (117.2 ± 13.04 pg/mL) were strongly elevated in plasma PBMCsec, while being undetectable in controls (Figure 4B). In contrast, several proinflammatory factors, such as IL-1β, TNF-α, and IFN-γ were not detectable in the plasma of PBMCsec-treated whole blood (data not shown). We next aimed to assess the proangiogenic capacity of fresh plasma, plasma of untreated whole blood, and plasma of PBMCsec-treated whole blood in an in vitro endothelial cell tube formation assay (Figure 4C). While controls showed little ability to induce tube formation, plasma of PBMCsec-treated whole blood displayed the highest proangiogenic properties as indicated by a significant increase in total segment length, as well as in numbers of nodes and junctions (Figure 4D). In summary, we could demonstrate that the expression and secretion of proangiogenic paracrine factors were strongly increased in whole blood cells following treatment with PBMCsec.

### 3.4. PBMCsec Inhibits Protease Activity In Vitro and Induces the Selective Serine Protease SERPINB2 in Human Whole Blood Ex Vivo

**Figure 5:**
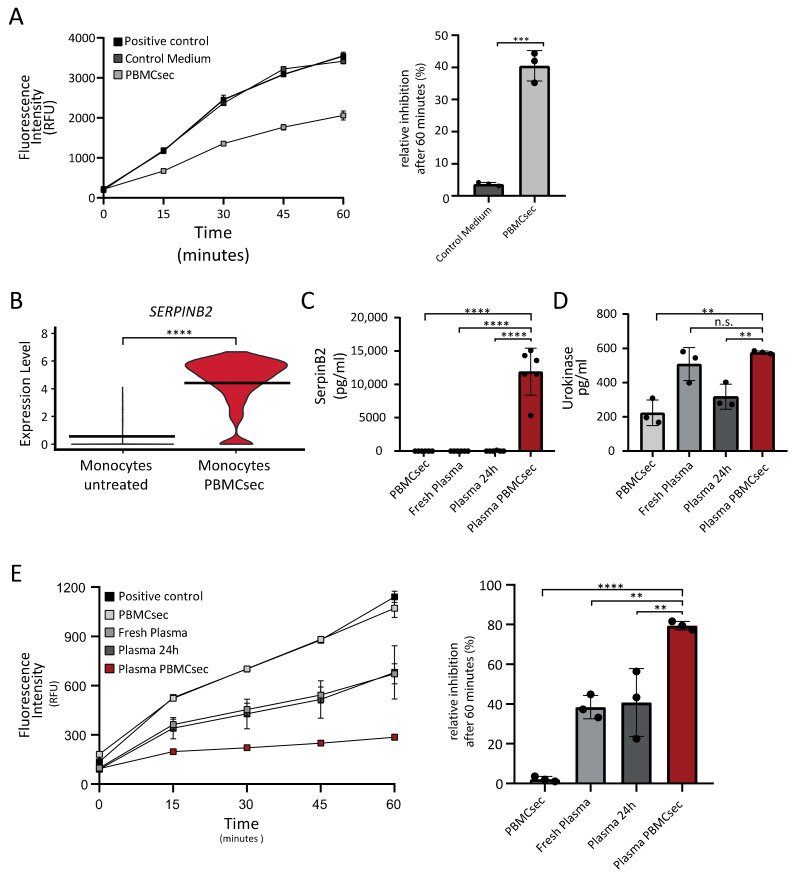
PBMCsec inhibits serine protease activity in vitro and increases levels of SERPINB2 in plasma of PBMCsec-treated whole blood. (**A**) Trypsin activity measured by increase in fluorescence intensity of cleaved substrate over time in the presence and absence of PBMCsec (*n* = 3 biological replicates). (**B**) Gene expression of the urokinase inhibitor *SERPINB2* in monocytes treated with PBMCsec (*p*-value < 0.0001) compared to untreated monocytes. (**C**) SerpinB2 (*n* = 3 biological replicates measured in duplicates) and (**D**) urokinase (*n* = 3 biological replicates) protein levels of fresh plasma, untreated plasma, and plasma of PBMCsec-treated whole blood. (**E**) In vitro assessment of urokinase activity of fresh plasma, plasma from untreated whole blood, and plasma obtained from PBMCsec-treated whole blood. ** *p* < 0.01, *** *p* < 0.001, **** *p* < 0.0001.

Increased protease activity, as seen in acute cardiovascular or inflammatory diseases, has been shown to significantly contribute to tissue destruction, thus representing an attractive target for tissue regeneration [42,43]. Evidence from previous studies identified monocytes as a source of protease inhibitors [44]. As our pathway analysis also revealed an association of upregulated genes in monocytes with the regulation of endopeptidase activity, we next aimed to confirm this finding in functional assays in vitro. Therefore, we performed a protease activity assay to test a potential anti-proteolytic effect of PBMCsec on the nonselective serine protease trypsin. While the medium control showed a negligible inhibitory effect on protease activity (3.72 ± 0.55%), PBMCsec led to a significant inhibition of the enzymatic activity (40.51 ± 4.728%) (Figure 5A). Furthermore, SERPINB2 displayed the highest positive log_2_ fold-change induction (Figure 5B) of all DEGs in monocytes treated with PBMCsec in our sequencing analysis. SERPINB2 is a member of the serpin superfamily of serine proteases and encodes plasminogen activator inhibitor type II (PAI-2), which is involved in the irreversible inhibition of urokinase [45]. First, we determined the protein levels of SERPINB2 in PBMCsec, fresh plasma, plasma of untreated whole blood, and then compared them to plasma of PBMCsec-treated whole blood. In accordance with the present literature, only very low levels of SERPINB2 were detectable in the control samples. In contrast, plasma obtained from PBMCsec-treated whole blood showed significantly elevated levels of SERPINB2 (11908 ± 3530 pg/mL) (Figure 5C). As SERPINB2 mainly acts as an inhibitor for urokinase [45], we next evaluated the plasma levels of urokinase, as well as the inhibitory effect of plasma PBMCsec on its activity in vitro. Whereas high levels of urokinase were still detected (Figure 5D), urokinase activity was strongly reduced by PBMCsec-treated plasma (Figure 5E), indicating that the observed urokinase inhibitory action was a result of the presence of a urokinase inhibitor rather than a quantitative decrease in available urokinase. While in vitro urokinase activity was not affected by PBMCsec alone, addition of fresh plasma (38.45% ± 5.94%) and plasma of untreated whole blood (40.78% ± 17.07%) resulted in a considerable inhibition of urokinase activity. Interestingly, this effect was even more pronounced in the presence of plasma obtained from PBMCsec-treated whole blood (79.48 ± 2.15%) (Figure 5E). Together, these data show that soluble factors with anti-proteolytic activities were present in PBMCsec or strongly induced in white blood cells after treatment with PBMCsec.

### 3.5. PBMCsec Ameliorates Thrombin-Induced Decrease in Endothelial Barrier Function

**Figure 6:**
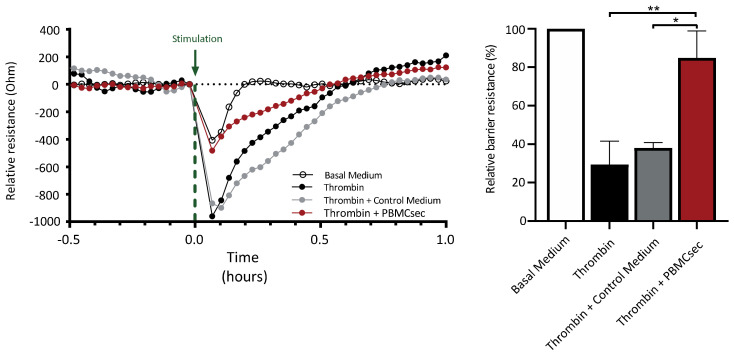
PBMCsec ameliorated the thrombin-induced decrease in endothelial cell barrier function. Representative evaluation of one of three independently conducted experiments. Paracellular resistance at 250 Hz was measured in DMECs challenged with 2 units/mL thrombin alone or in combination with control medium (CellGenix) or PBMCsec at 12.5 units/mL over time. The green arrow marks the time point of stimulation with the respective treatments. The bar plot shows the changes in endothelial barrier function for the investigated conditions relative to the basal medium (EBM-2) control. One-way ANOVA was performed with post hoc Dunnett’s multiple comparison test to compare groups to basal medium; * *p* < 0.05, ** *p* < 0.01.

The activity of inflammatory mediators and serine proteases has been shown to support angiogenesis, as well as increase vascular leakage, by decreasing the endothelial barrier function [38,46]. Electrical cell-substrate impedance sensing enables the continuous assessment of changes in quality and function of a cellular barrier in response to different stimuli over time [47]. Loss of barrier function in endothelial cells can result in increased extravasation of immune cells and harmful mediators into the extravasal space, further increasing tissue damage [48]. Hence, we examined whether PBMCsec affected the thrombin-induced drop in endothelial barrier function by utilizing electrical cell-substrate impedance sensing. Addition of thrombin resulted in a transient decrease in trans-endothelial resistance, reaching the lowest point 5 min after stimulation (29% ± 12% barrier function relative to untreated basal medium control) (Figure 6). While the simultaneous addition of thrombin and control medium resulted in a similar change in barrier resistance (38% ± 2%) compared to basal medium, PBMCsec inhibited the thrombin-induced decrease in endothelial resistance (84% ± 14%) (Figure 6). Therefore, PBMCsec positively influenced endothelial barrier function in vitro by attenuating thrombin-mediated changes in endothelial cells.

## 4. Discussion

The beneficial effects of PBMC-derived cell secretomes in the regeneration of damaged tissues and organs have already been well described [10,15,21,24,25]. In order to transit from these promising preclinical studies to the treatment of patients, the safety and tolerability of topical administration of autologous PBMCsec in dermal wounds was successfully assessed in a clinical phase I trial (MARSYAS I, NCT02284360) [49]. On the basis of these results, an ongoing clinical phase I/II trial has been initiated to evaluate the safety and efficacy of topically administered allogeneic PBMCsec for the treatment of diabetic foot ulcers (MARSYAS II, NCT04277598) [50]. In light of future therapeutic approaches using systemic administration of PBMCsec to regenerate inner organs, we analyzed in the present study the effects of PBMCsec on whole blood and the endothelium in more detail.

Using scRNA-seq, we identified a strong induction of tissue-regenerative pathways after treatment of human whole blood with PBMCsec ex vivo. In particular, biological processes associated with the regulation of vasculature development, wound healing, and regulation of endopeptidase activity, as well as decreased superoxide anion generation and leukocyte degranulation, all of which contribute to tissue regeneration, were significantly overrepresented in our analysis. The observed changes of the transcriptional profile of white blood cells treated with PBMCsec were most pronounced in monocytes, while all other cell types showed comparably low alterations in gene expression. Our finding is in line with previous publications where functional changes were mainly reported in monocytes after stimulation with conditioned medium from MSCs [51,52]. As monocytes are an important part of the innate immune system, a rapid response to extracellular stimuli, such as PBMCsec, was expected. Interestingly, only few granulocytes, which also represent a main constituent of innate immunity, were detected in our single-cell analysis. A low detectability of the different granulocyte populations in scRNA-seq has been reported before and might be due to their overall low RNA content and presence of RNAses [53]. This assumption is further supported by the extremely low mRNA counts in the few granulocytes detected in our analysis. Interestingly, T- and B-cells also showed only minor gene regulation after exposure to the secretome. As both cell types belong to the adaptive branch of the immune system, it is tempting to speculate that repeated exposure of lymphoid cellular subsets to PBMCsec might result in a more pronounced adaptive immune response. Furthermore, the natural process of aging may lead to various changes in immune cells from whole blood, which manifest as decreased immunological activity in older individuals. Indeed, previous studies in animal models have reported on positive age-associated effects of blood from young donors [54]. However, in light of our ongoing and future clinical studies, we here used PBMCsec produced under GMP conditions, which represents a pool of donors in the age range of 18–45 years. As pooling averages all active factors in PBMCsec, further studies will be necessary to fully address age-related effects.

Examination of the altered gene sets in monocytes revealed upregulation of several members of the chemokine (C–X–C motif) ligand (CXCL) family (*CXCL1*, *CXCL2*, *CXCL3*, *CXCL5*, and *CXCL8*), as well as different growth factors after stimulation with PBMCsec. In addition, high protein levels of CXCL1, CXCL5, and VEGF-A were detected in the plasma of PBMCsec-treated whole blood. These factors have previously been described as important constituents of PBMCsec, contributing to its tissue-regenerative actions [13,15]. Yet, we were not able to detect these proteins in PBMCsec in our assays. This discrepancy could be explained by the lower concentration of PBMCsec that we used in our ex vivo study. The lower concentration of PBMCsec had to be used for the stimulation of PBMCs, and endothelial cells as 1:2 dilutions with fresh medium were necessary for cell viability. Nevertheless, all these factors were strongly induced in whole blood and abundantly present in the plasma derived from PBMCsec-treated whole blood, indicating a significant amplification of the production of tissue regenerative factors. In addition, our transcriptional analysis revealed a downregulation of genes involved in the generation of superoxide anion species with an inhibitory effect on the formation of reactive oxygen species in PBMCsec-treated monocytes. Expression of the scavenger receptor CD36 on monocytes treated with PBMCsec was amongst the most strongly downregulated genes. In monocytes and macrophages, activation of this receptor promotes increased production of reactive oxygen species, which in turn reinforced damage to the vasculature [55]. Furthermore, CD36 is known to modulate the uptake of oxidized lipid species by macrophages, leading to functional changes in these cells. As they accumulate lipids, these so-called foam cells then promote proliferation and inflammation of endothelial and smooth muscle cells, which contributes to the formation of atherosclerotic plaques [56]. Given that reactive oxygen species increase after re-establishment of perfusion and aggravate tissue damage in a series of cardiovascular pathologies, our data offer a potential mechanism via which PBMCsec might directly and/or indirectly reduce tissue damage after ischemic conditions. Future studies will elucidate the influence of PBMCsec on the generation and amelioration of reactive oxygen species.

Various biological active components have already been identified, contributing to the diverse effects of PBMCsec, including cytokines, growth factors, lipids, and exosomes. In particular, lipids present in PBMCsec have been shown to strongly impact immune cell functions. Laggner et al., recently demonstrated that PBMCsec-derived lipids interfere with dendritic cell differentiation and mast cell and basophil granulocyte degranulation, thereby improving skin inflammation and allergic symptoms, respectively [19,20]. This special composition of biologically active cytokines, growth factors, lipids, and exosomes, amongst other yet unidentified substances, makes PBMCsec a highly effective compound against tissue damage and inflammation. Previous work from our group demonstrated that paracrine factors released from γ-irradiated PBMCs positively influence tissue regeneration by affecting endothelial cell survival and sprouting [12]. In addition, we here showed that PBMCsec was also able to inhibit thrombin-induced disruption of the endothelial barrier in dermal microvascular endothelial cells. This effect might counteract increased leakage of blood vessels, thereby preventing insufficient perfusion of the vital part of the injured organ and amplification of the damage. We also observed an inhibitory effect of the plasma of PBMCsec-treated whole blood on the serine protease urokinase, which was not detected in PBMCsec alone. Thus, de novo synthesis of the urokinase inhibitor SERPINB2 could mediate this effect. Serine protease inhibitors are widely discussed as promising therapeutic approaches for several disease entities. Recently, Vorstandlechner et al. identified dipeptidyl-peptidase 4 (DPP4) and urokinase (PLAU) as important drivers of skin fibrosis, and targeted inhibition of both molecules was shown to reduce myo-fibroblast formation and improve scar quality [57]. Implications for therapeutic targeting of serine proteases are also evident for myocardial infarction [58,59]. While early re-vascularization of occluded blood vessels still remains the gold-standard intervention to maximize rescue of functional tissue, increasing emphasis is being put on the reduction of late onset events caused by accumulation of proinflammatory stimuli, proteases, and other detrimental factors, secondary to reperfusion injury [32]. Mauro and colleagues reported reduced infarction sizes following treatment with alpha-1-antitrypsin, in addition to revascularization, in a murine model of myocardial infarction [60]. This was further reinforced by a report by Hooshdaran et al., where inhibition of the serine proteases cathepsin G and chymase was shown to reduce adverse cardiac remodeling, myocyte apoptosis, and fibrosis in a murine model of myocardial ischemia reperfusion injury [61]. In addition, a recent publication by Sen and colleagues reported a central role for SerpinB2 in the coordinated resolution and repair of damaged tissue after ischemia/reperfusion in a murine kidney injury model [62]. These data suggest that, in addition to the protective action of serine protease inhibitors investigated in our study, they might also show valuable tissue-regenerative and antifibrotic properties. Further studies are needed to fully decipher the contribution of protease inhibitors already present in PBMCsec, as well as de novo induced inhibitors such as SerpinB2, and their respective roles in the inhibition of tissue-damage or the restoration of damaged tissue and organs.

Paracrine signaling for the prevention of a proinflammatory response and cell death, as well as induction of angiogenesis, is essential to restore damaged tissues and organs [52,63]. We previously showed that PBMCsec was able to positively modulate all of these events [12,15,64], and administration of a single dose of PBMCsec was sufficient to almost completely inhibit heart damage in a porcine model of experimental myocardial infarction [15]. However, given that pharmacodynamic investigations revealed that several main components of PBMCsec were only traceable for minutes to a maximum of 5 h in the blood of rats and dogs after intravenous application of human PBMCsec, this finding was rather unexpected [65]. Whether human PBMCsec induces a comparable release of pro-regenerative factors in these animal models has not been investigated so far. Our new data provide a reasonable explanation for this observation and suggest that PBMCsec induces the production of a new secretome in human white blood cells with additional regenerative properties. This is in line with our findings of increased endothelial cell sprouting after treatment of HUVECs with plasma of PBMCsec-treated whole blood. Cell types other than PBMCs, which could also contribute to the regenerative effects, can be found in whole blood. Thrombocytes are known for their proangiogenic properties, which are mainly due to the release of VEGF upon thrombocyte activation [66]. Interestingly, previous work from our group identified an inhibitory effect of PBMCsec on platelet activation and aggregation [18], suggesting that PBMCsec does not promote the release of proangiogenic mediators from thrombocytes. In contrast, an increased activity was observed in untreated plasma after cultivating whole blood for 24 h, as compared to fresh plasma, which might be a result of thrombocyte activation in the absence of PBMCsec. Importantly, our results indicate that the pharmacodynamic effect of a single application of PBMCsec can be significantly prolonged by continuous stimulation, comparable to the behavior of a damped wave. Therefore, this induced secretome, produced over an extended period of time, might multiply the pro-regenerative properties of PBMCsec by combining them with those of the newly induced factors. However, further studies in a human setting will be necessary to fully explore the tissue-regenerative potential of the newly formed secretome and to examine for how long this effect can be maintained.

In summary, our scRNA-seq analysis identified key mechanisms, potentially contributing to tissue regeneration, which might occur after systemic application of a cell secretome derived from irradiated PBMCs. PBMCsec displays a broad spectrum of mechanistic modes of actions that greatly complement each other and, therefore, positively contribute to tissue regeneration in a wide range of pathological settings. In addition, our data suggest that the effects in vivo are not restricted to direct actions of PBMCsec but also arise from further stimulation of circulating monocytes in the blood. Further human clinical studies in the future will clarify the underlying mechanisms and the therapeutic benefit of systemic treatment of damaged organs with PBMC-derived secretomes.

## 5. Patents

The Medical University of Vienna has claimed financial interest. H.J.A. holds patents related to this work (WO 2010/079086 A1; WO 2010/070105 A1; EP 3502692; European Patent Office application #19165340.1).

## Data Availability

ScRNA-seq data are available upon request.

## Supplementary Materials

The following supporting information can be downloaded at https://www.mdpi.com/article/10.3390/pharmaceutics14081600/s1: Figure S1. Identification of cell clusters based on expression of cluster-defining marker genes for T-cells, B- cells, monocytes, and granulocytes; Figure S2. Donor comparability of detected cell types and identification of pathways associated with upregulated genes in monocytes treated with PBMCsec; Figure S3. Transcriptional changes in T-cells, B-cells, and granulocytes and their predicted regulation of biological processes; Figure S4. Biological processes affected by up- and downregulated genes in monocytes treated with PBMCsec; Figure S5. Downregulated genes in monocytes treated with PBMCsec associated with leukocyte degranulation and generation of reactive oxygen species; Supplementary sheet 1. Pathways associated with significantly upregulated genes in monocytes treated with PBMCsec; Supplementary sheet 2. Pathways associated with significantly downregulated genes in monocytes treated with PBMCsec.
