# Supplementary material for: Paracrine Factors of Stressed Peripheral Blood Mononuclear Cells Activate Proangiogenic and Anti-Proteolytic Processes in Whole Blood Cells and Protect the Endothelial Barrier
Source: Pharmaceutics. 2022 Jul 30;14(8):1600. doi: 10.3390/pharmaceutics14081600 (PMC9415091; doi:10.3390/pharmaceutics14081600)

A

leukocyte degranulation and regulation of cell activation

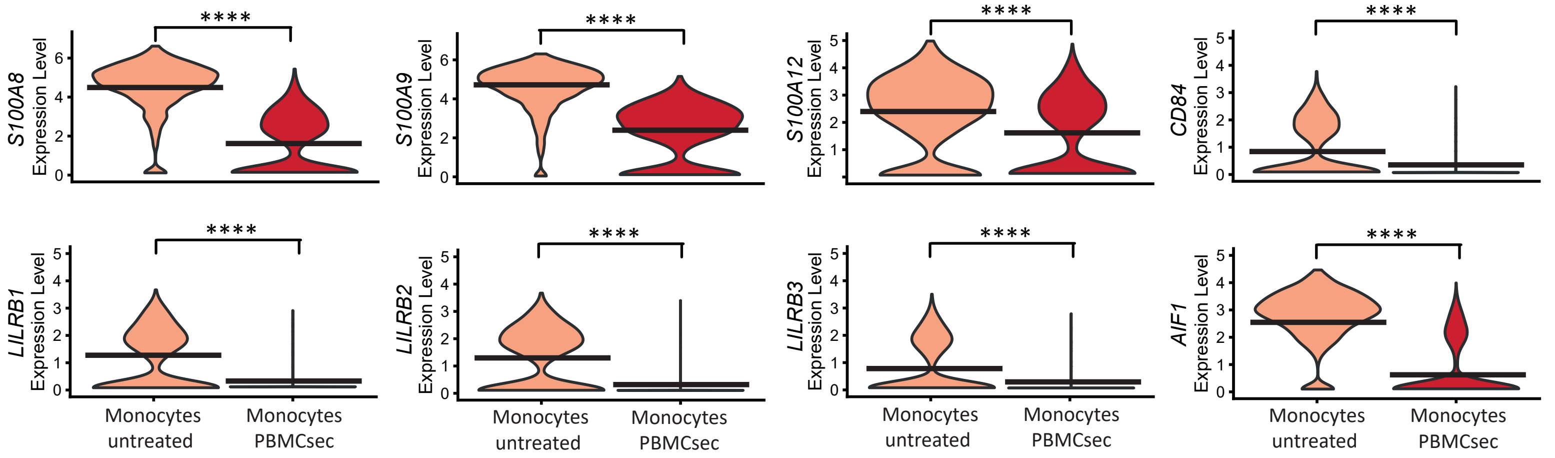


B

superoxide anion generation and response to reactive oxygen species

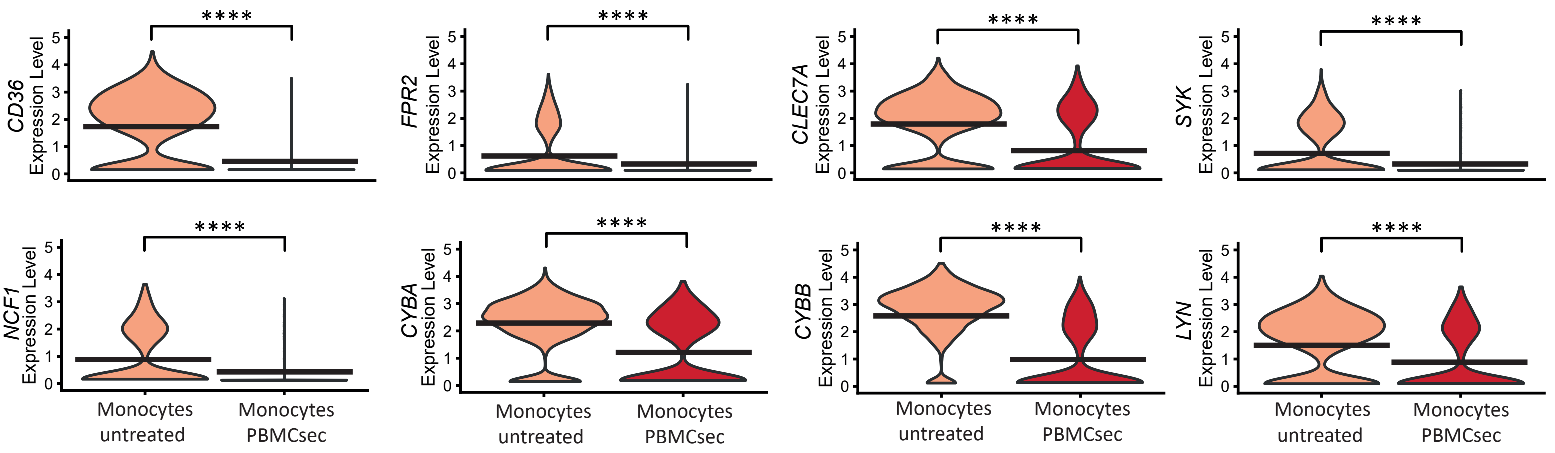

Supplement: Supplementary file 1 [file pharmaceutics-14-01600-s001.zip › Figure_S5.pdf]
