# Supplementary material for: Paracrine Factors of Stressed Peripheral Blood Mononuclear Cells Activate Proangiogenic and Anti-Proteolytic Processes in Whole Blood Cells and Protect the Endothelial Barrier
Source: Pharmaceutics. 2022 Jul 30;14(8):1600. doi: 10.3390/pharmaceutics14081600 (PMC9415091; doi:10.3390/pharmaceutics14081600)
Supplement: Supplementary file 1 [file pharmaceutics-14-01600-s001.zip › Supplemental Information Copic et al.pdf]

Supplemental Information

Paracrine factors of stressed peripheral blood mononuclear cells activate pro-angiogenic and anti-proteolytic processes in whole blood cells and protect the endothelial barrier

Dragan Copic[1,2], Martin Direder[1,2], Klaudia Schossleitner[3], Maria Laggner[1,2], Katharina Klas[1,2], Daniel Bormann[1,2], Hendrik Jan Ankersmit,[1,2, §, *] and Michael Mildner,[4, §, *]

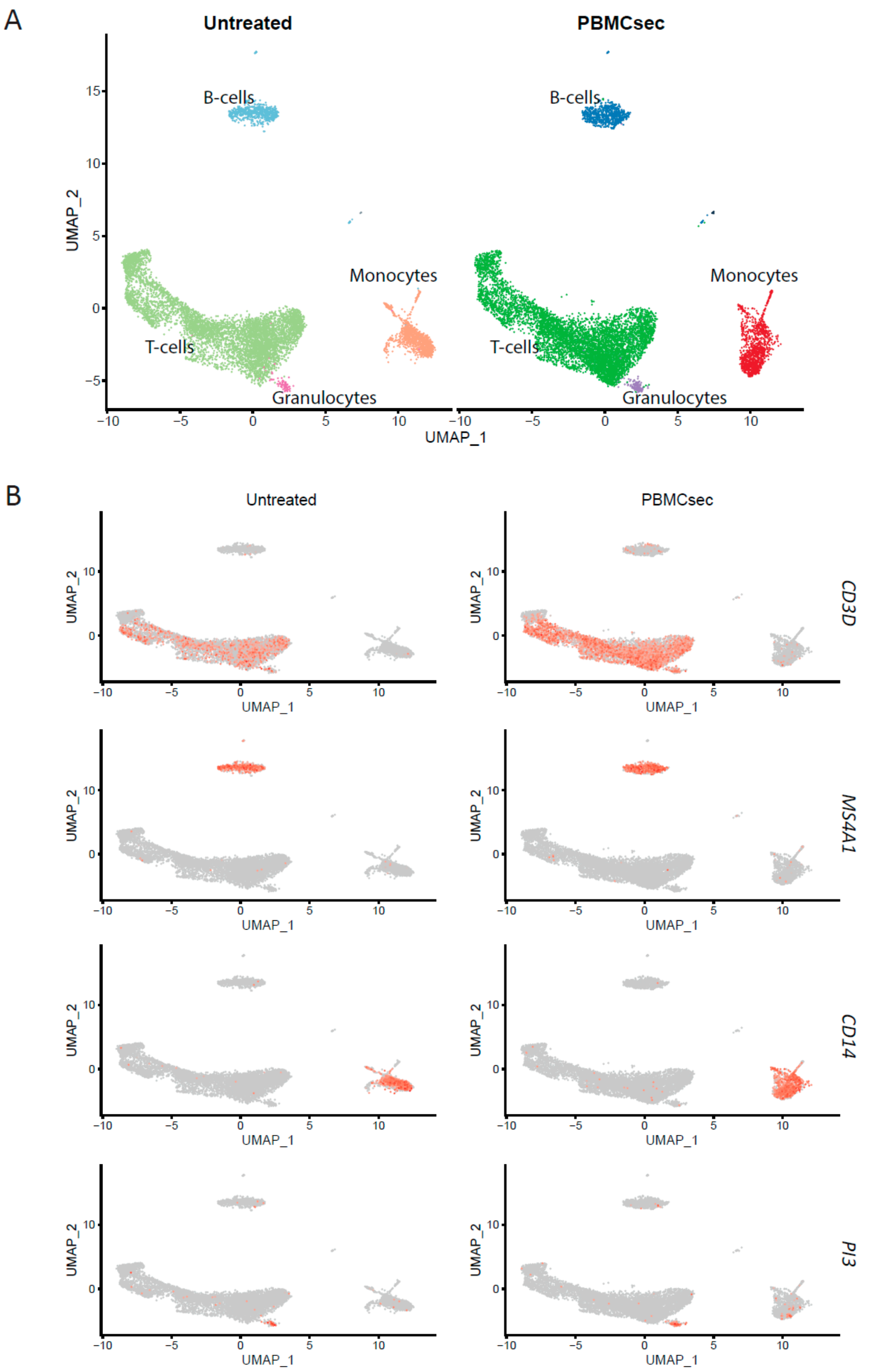

Supplementary Figure S1. Identification of cell clusters based on expression of cluster-defining marker genes for T-cells, B- cells, Monocytes and granulocytes.
(A) UMAP Plot with cell clusters and (B) Featureplots showing the average expression for *CD3D*, *MS4A1*, *CD14* and *PI3* in every cluster. Positive events for the respective marker gene are highlighted in red. Identified clusters are annotated as T-cells, B-cells, monocytes and granulocytes.

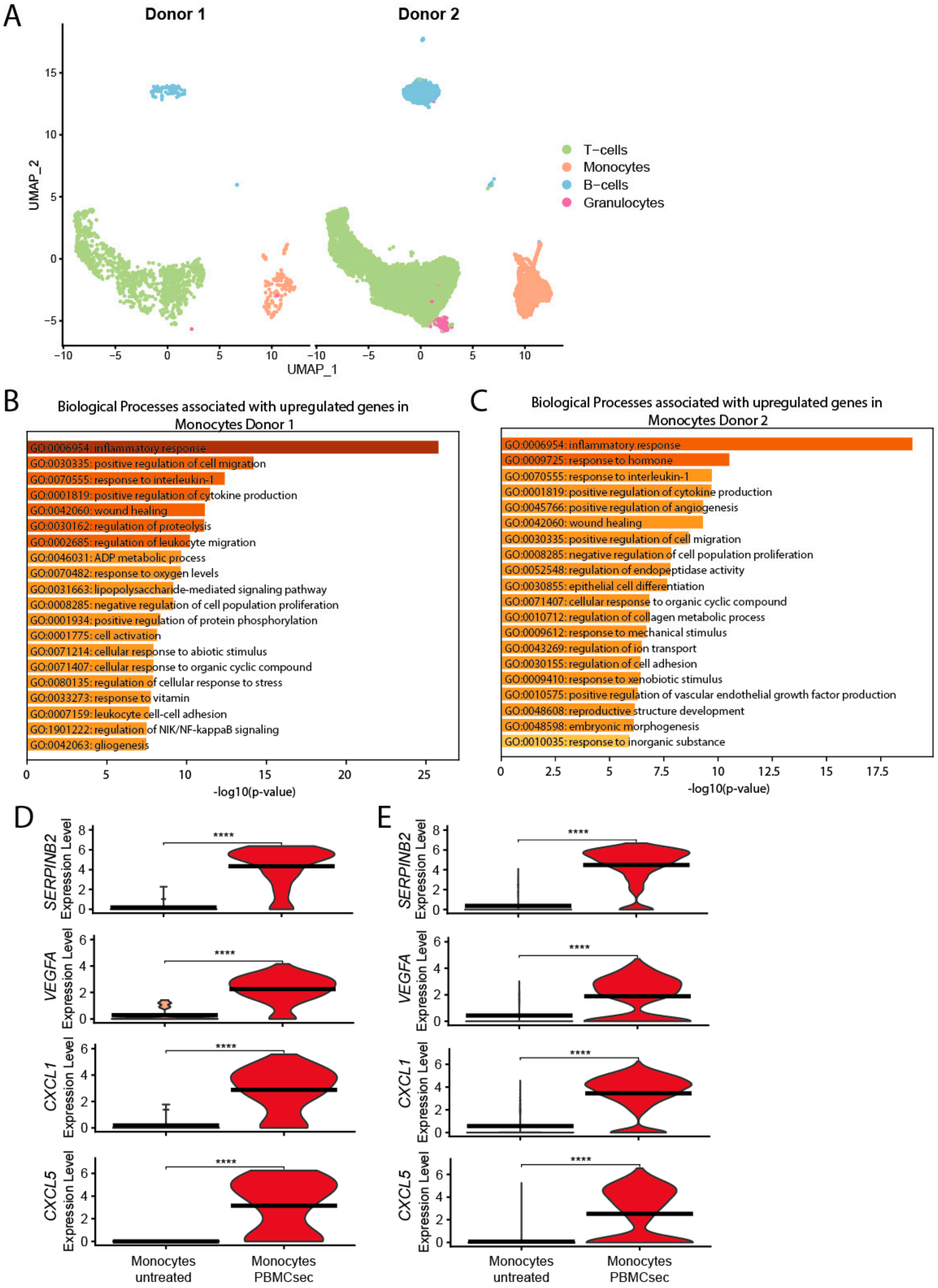

Supplementary Figure S2. Donor comparability on detected cell types and identification of pathways associated with up-regulated genes in monocytes treated with PBMCsec.

(A) UMAP Plot with cell clusters split by donors. Pathway analysis for significantly regulated biological processes in monocytes treated with PBMCsec for (B) donor 1 and (C) donor 2.

Violinplot showing the average expression for *SERPINB2*, *VEGFA*, *CXCL1* and *CXCL5* in monocytes (untreated vs. PBMCsec) for (D) donor 1 and (E) donor 2. **** indicate p-value < 0.0001.

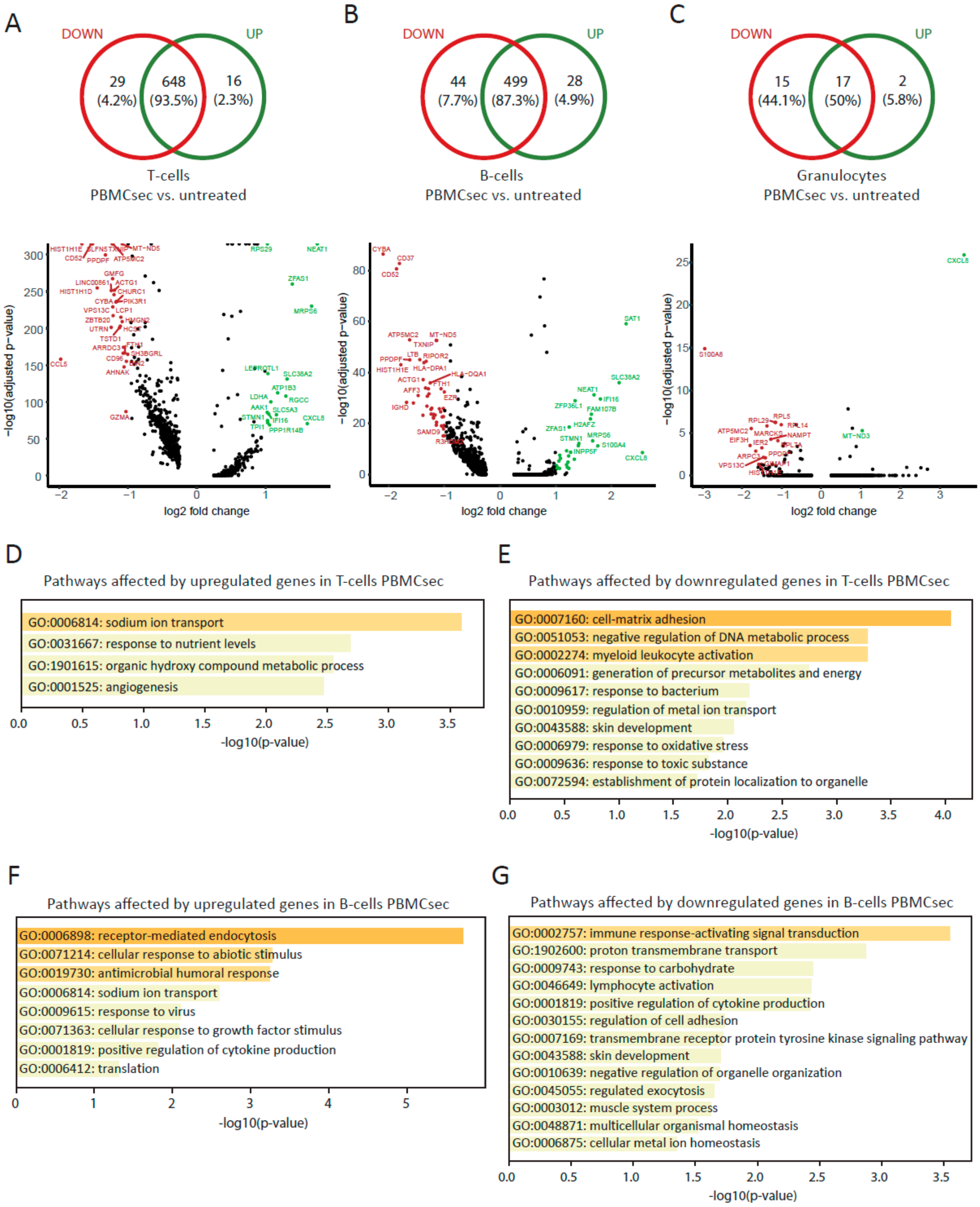

Supplementary Figure S3. Transcriptional changes in T-cells, B-cells and granulocytes and their predicted regulation of biological processes.

Distribution of up- and downregulated genes are shown for (A) T-cells, (B) B-cells and (C) granulocytes. Barplots display the significantly enriched biological processes affected by (D) upregulated and (E) downregulated gene sets for T-cells. Barplots display the significantly enriched biological processes affected by (F) upregulated and (G) downregulated gene sets for B-cells.

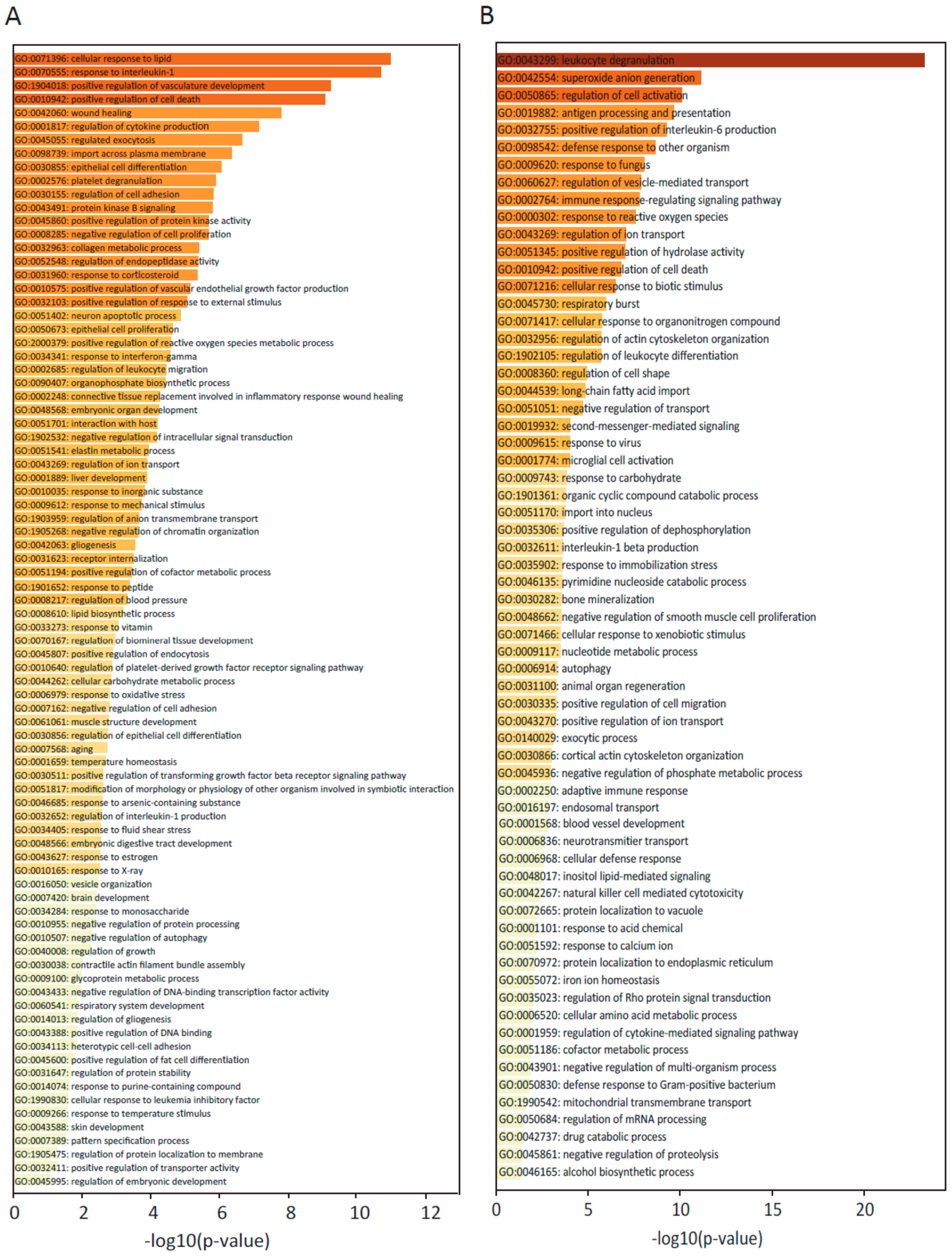

Supplementary Figure S4. Biological processes affected by up- and downregulated genes in monocytes treated with PBMCsec.

(A) Barplot of significantly biological processes enriched by the the gene set of upregulated genes and (B) downregulated genes in monocytes treated with PBMCsec.

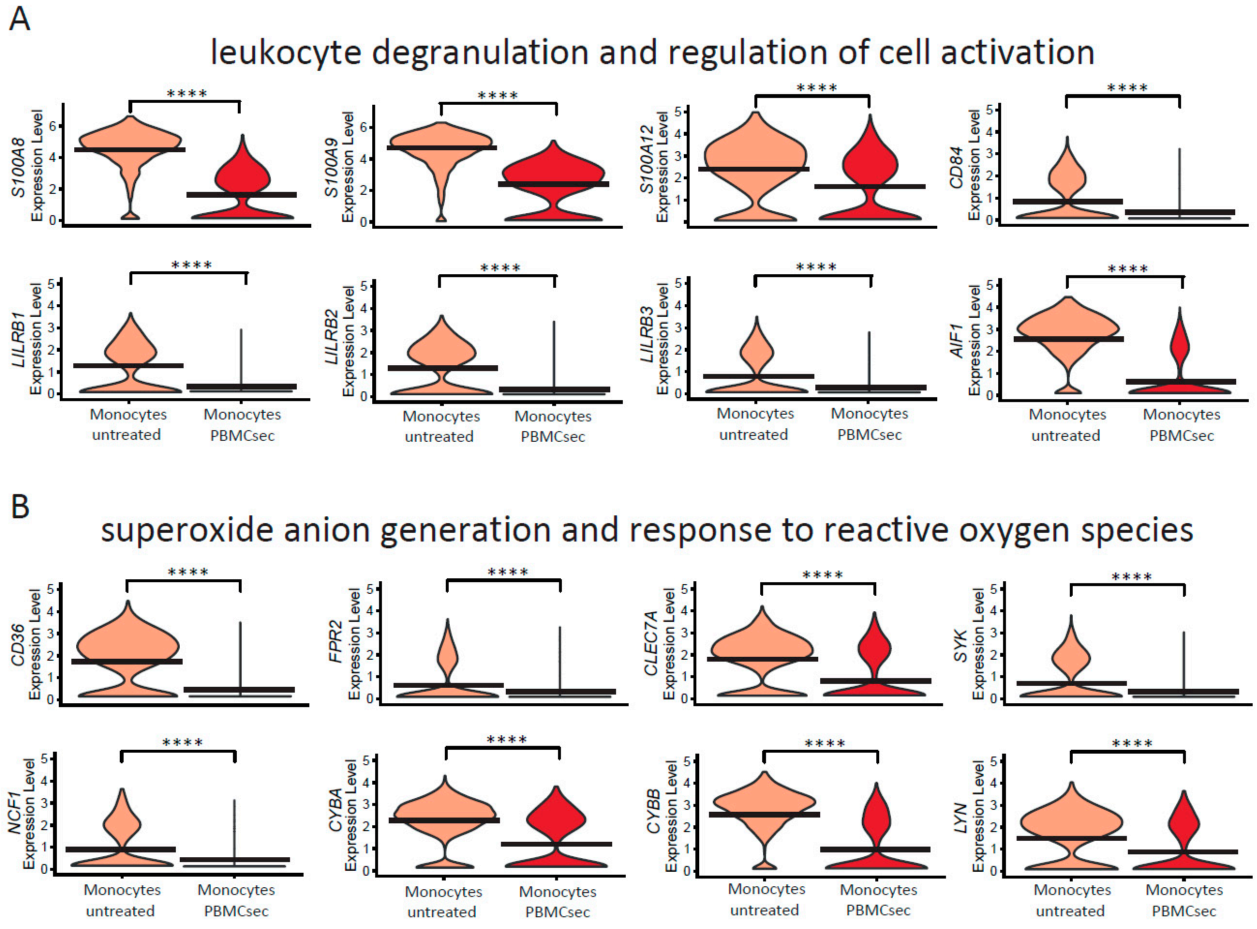

Supplementary Figure S5. Downregulated genes in monocytes treated with PBMCsec associate with leukocyte degranulation and generation of reactive oxygen species. (A) Violin Plots show a selection of most downregulated genes that contribute to leukocyte degranulation and activation and (D) superoxide anion generation and response to reactive oxygen species. **** indicate p-value < 0.0001.

Supplementary sheet S1. Pathways associated with significantly upregulated genes in monocytes treated with PBMCsec.

Supplementary sheet S2. Pathways associated with significantly downregulated genes in monocytes treated with PBMCsec.
