# Supplementary figures and images for: Paracrine Factors of Stressed Peripheral Blood Mononuclear Cells Activate Proangiogenic and Anti-Proteolytic Processes in Whole Blood Cells and Protect the Endothelial Barrier
Source: Pharmaceutics. 2022 Jul 30;14(8):1600. doi: 10.3390/pharmaceutics14081600 (PMC9415091; doi:10.3390/pharmaceutics14081600)

**A**

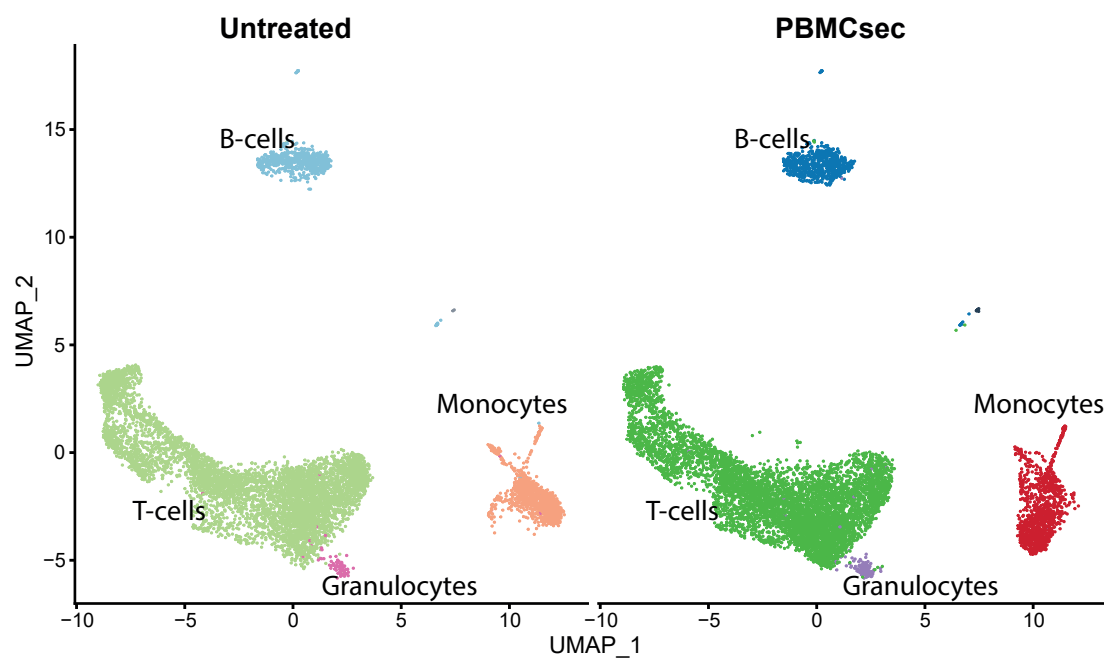

**B**

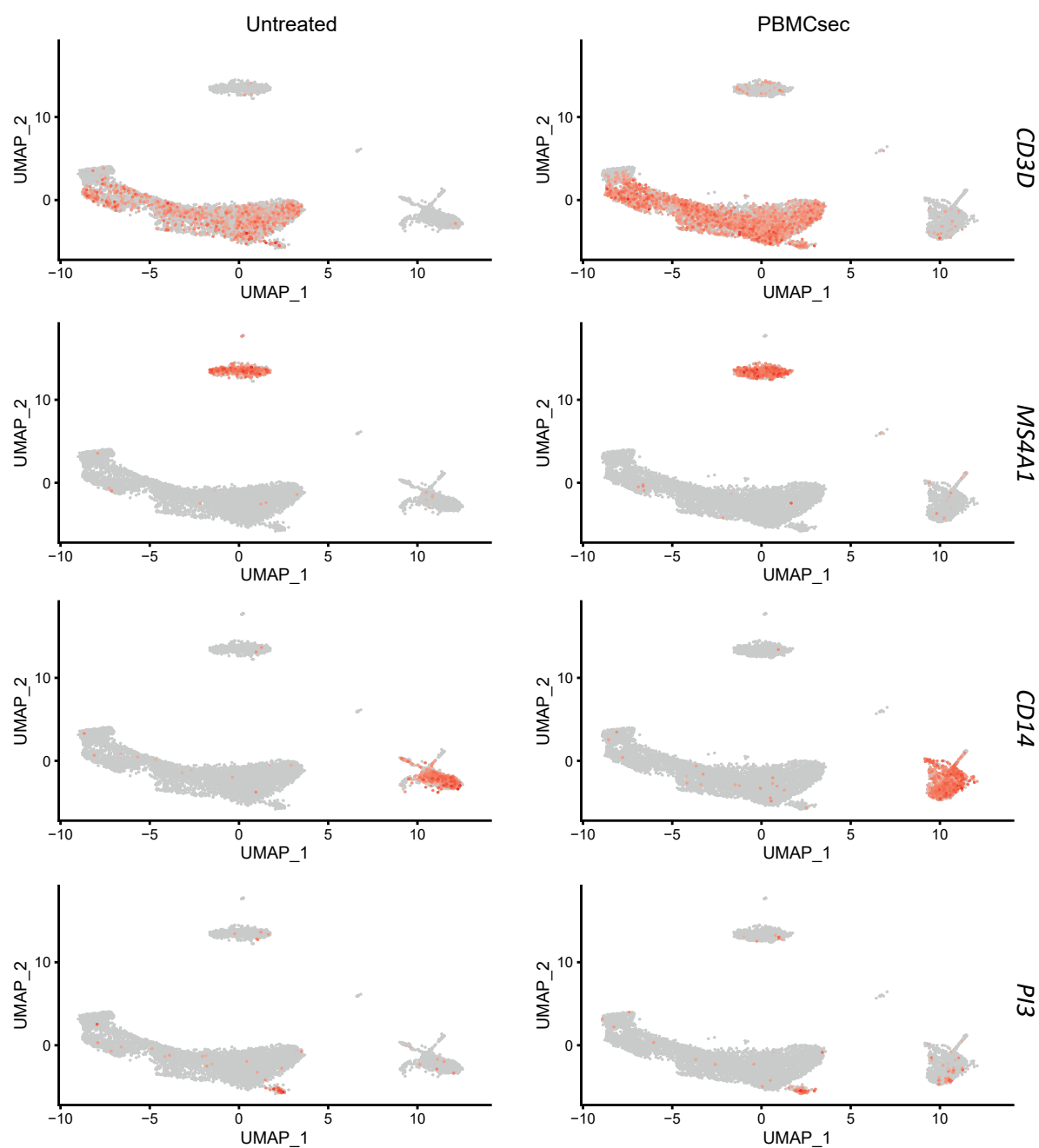

Supplement: Supplementary file 1 [file pharmaceutics-14-01600-s001.zip › Figure_S1.pdf]

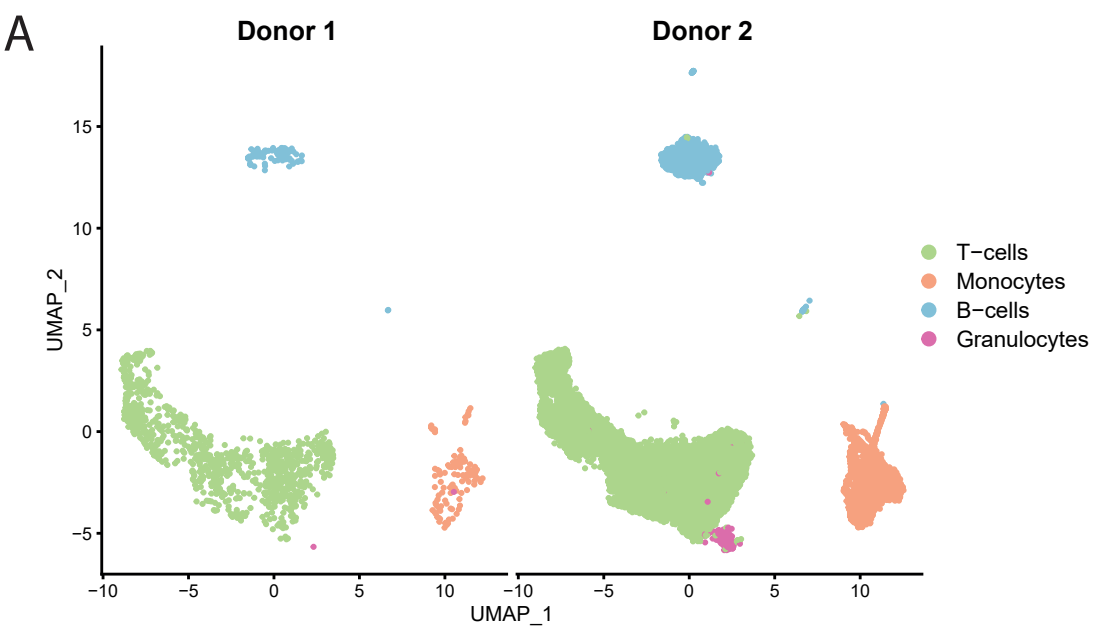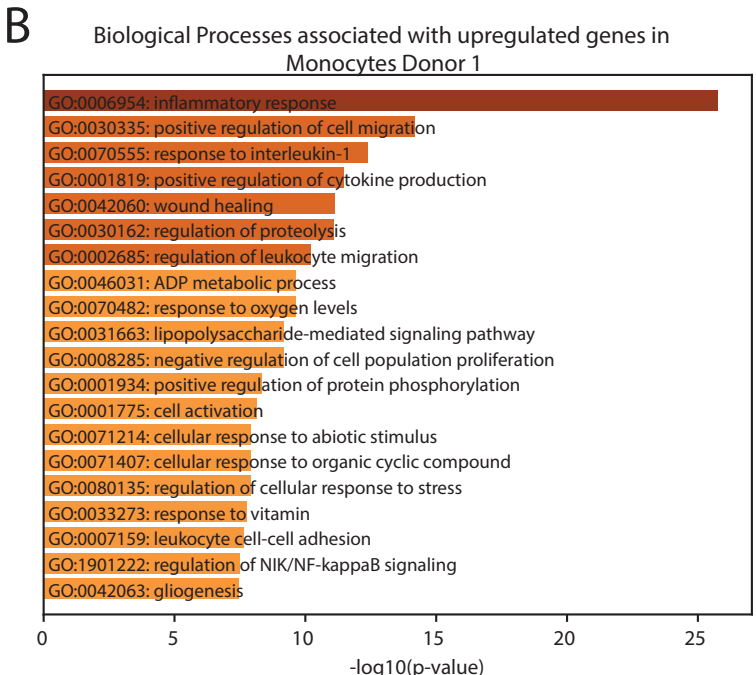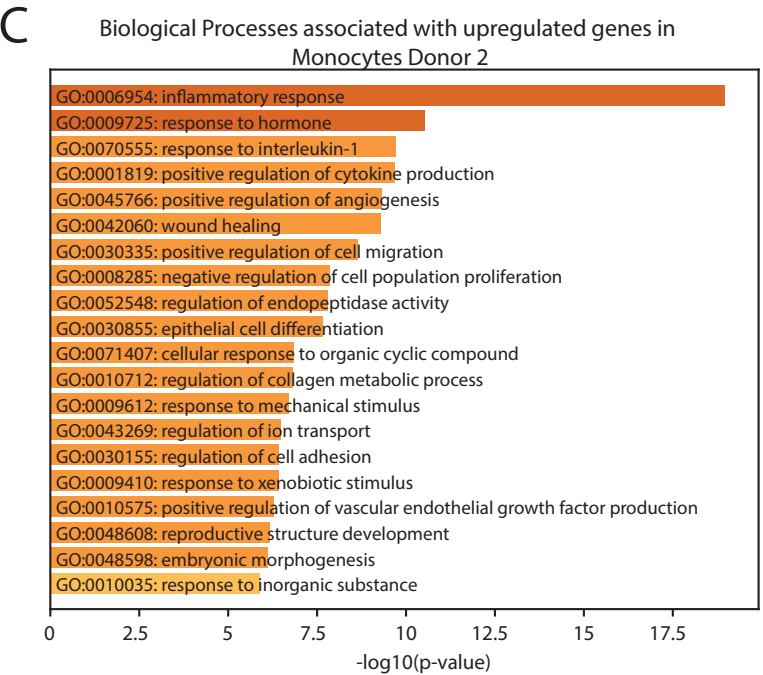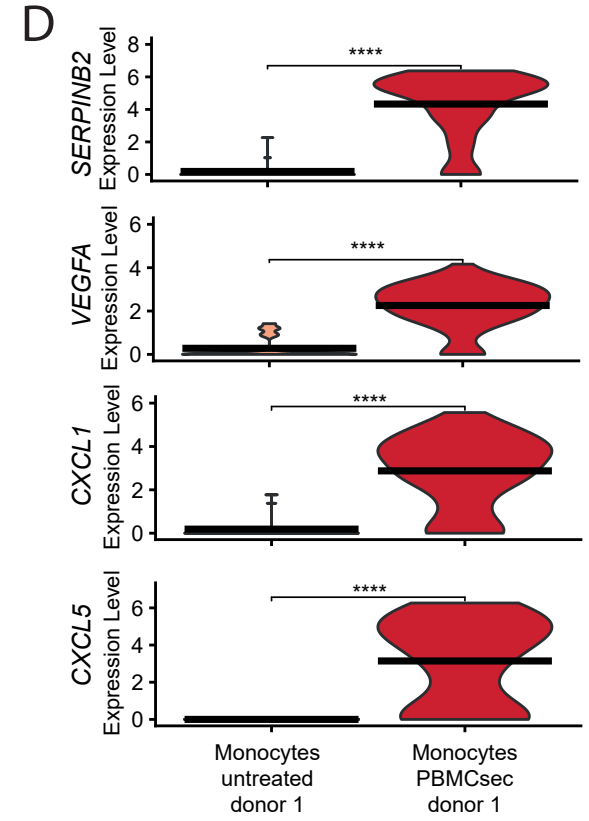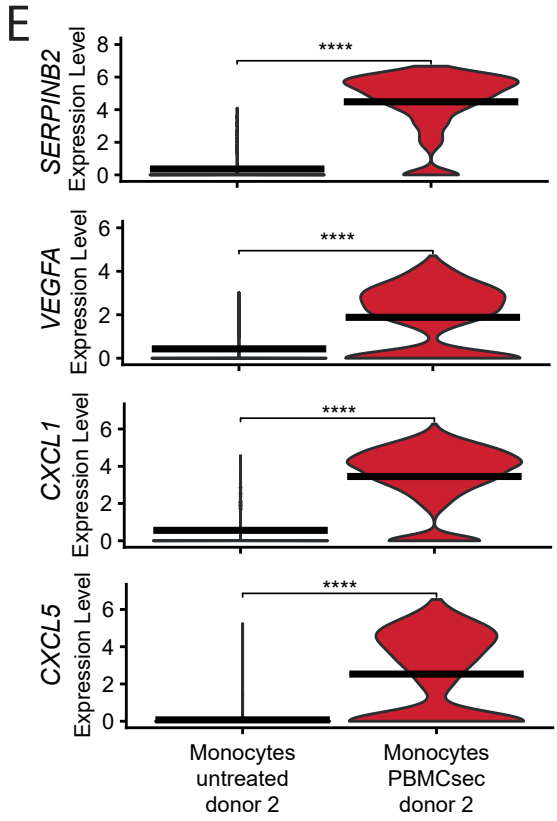

Supplement: Supplementary file 1 [file pharmaceutics-14-01600-s001.zip › Figure_S2.pdf]

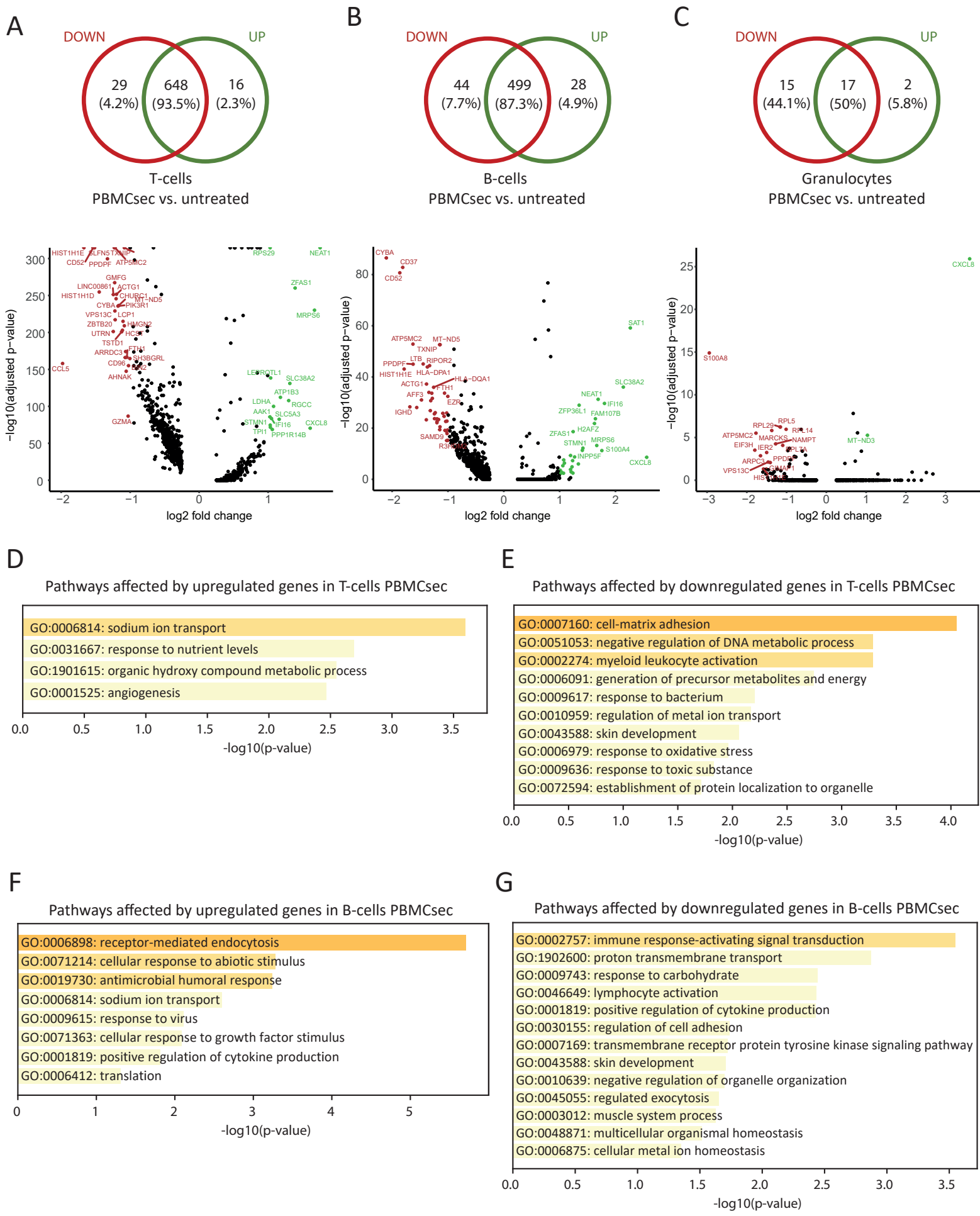

Supplement: Supplementary file 1 [file pharmaceutics-14-01600-s001.zip › Figure_S3.pdf]

A

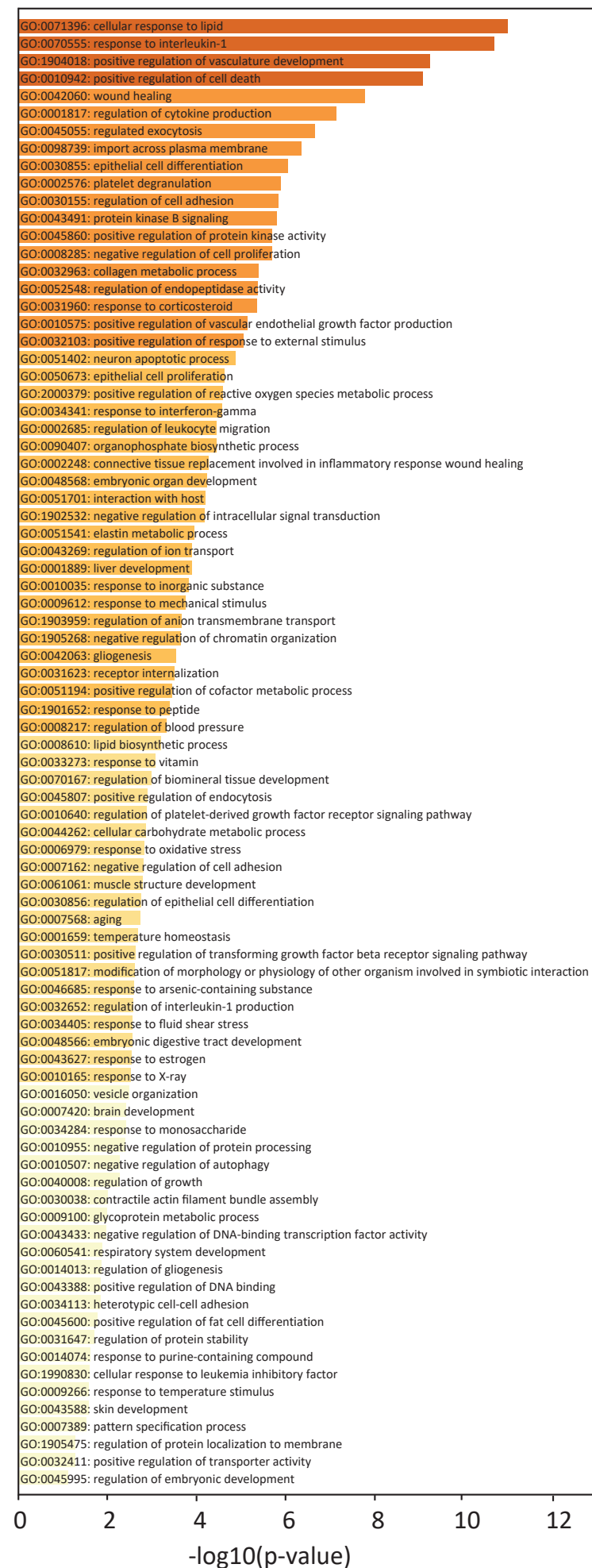

B

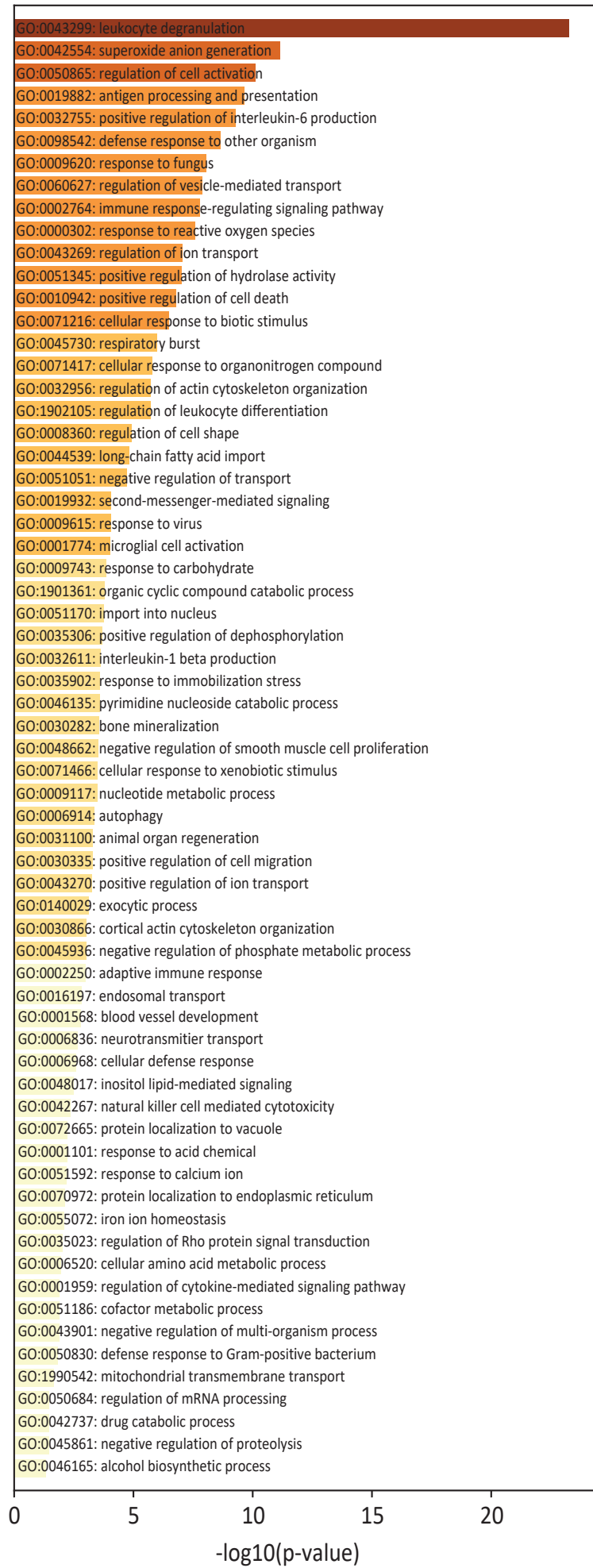

Supplement: Supplementary file 1 [file pharmaceutics-14-01600-s001.zip › Figure_S4.pdf]
